# Discovery of Cilnidipine Cocrystals with Enhanced Dissolution by the Use of Computational Tools and Semiautomatic High-Throughput Screening

**DOI:** 10.1021/acs.cgd.5c00184

**Published:** 2025-04-29

**Authors:** Matteo Guidetti, Rolf Hilfiker, Susan M. De Paul, Annette Bauer-Brandl, Fritz Blatter, Martin Kuentz

**Affiliations:** † Solid-State Development Department, 30275 Solvias AG, Römerpark 2, CH- 4303 Kaiseraugst, Switzerland; ‡ Department of Physics, Chemistry and Pharmacy, 6174 University of Southern Denmark, Campusvej 55, 5230 Odense, Denmark; § Institute of Pharma Technology, University of Applied Sciences and Arts Northwestern Switzerland, CH- 4132 Muttenz, Switzerland

## Abstract

Cocrystals are an attractive option for overcoming drug limitations, such as a low dissolution rate and absorption of poorly water-soluble compounds. Nevertheless, the discovery of new cocrystals remains a trial-and-error approach in which hundreds of coformers and several experimental methods are often tested. To streamline the cocrystal screening, computational methods can be used to select the coformers most likely to form a cocrystal, while high-throughput screening (HTS) approaches can rapidly screen them experimentally. In this manuscript, a new cocrystal of the extremely poorly soluble drug cilnidipine (solubility ≈30 ng/mL, 0.06 μM) was successfully discovered by applying HTS approaches. Only one cocrystal resulted from the screening with a total of 52 coformers, whereby the computational approach molecular complementarity successfully ranked this coformer (*p*-toluenesulfonamide) at the third position of the screening list. Dissolution studies conducted on the cocrystal in blank FaSSIF (fasted-state simulated intestinal fluid) and FaSSIF pH 6.5 revealed enhanced drug dissolution with a maximum achieved supersaturation equal to seven times the solubility of the crystalline drug. Dissolution rates of drug and coformer were compared for better mechanistic understanding of the cocrystal dissolution–supersaturation–precipitation behavior. The case of cilnidipine with a rare occurrence of cocrystals emphasized the importance of using joint computational and HTS approaches to enable successful cocrystal identification for pharmaceutical development.

## Graphical abstract

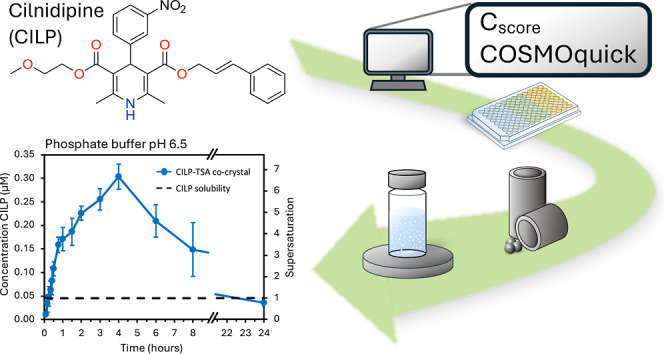

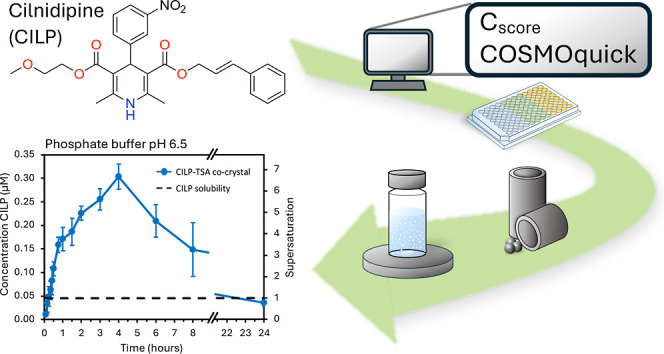

## Introduction

Most active pharmaceutical ingredients (APIs) in the development pipeline display low solubility in water and belong to Class II of the Biopharmaceutics Classification System (BCS).^1^ Poor aqueous solubility can lead to higher doses and erratic behavior when a drug is administered orally. A common strategy to address this issue is the formation of multicomponent solid forms, such as salts, cocrystals, and coamorphous systems.^2^

In particular, pharmaceutical cocrystals are developed by combining a poorly water-soluble API with a hydrophilic small organic molecule called a coformer. The two components are present in a defined stoichiometric ratio in a crystalline phase and, typically, are held together by nonionic interactions.^3^ The dissociation of the components in solution may generate supersaturation of the API, which is a driving force for molecular absorption and will eventually lead to the precipitation of the parent drug. Co-crystal dissolution, supersaturation, and precipitation (DSP) behavior can be modulated by changes in pH and surfactant concentration in the dissolution media^4,5^ or by addition of excess coformer.^6^

Despite the variety of experimental screening methods reported—including reaction crystallization,^7^ mechanochemical,^8^ and thermal^9^ methods—the discovery of cocrystals remains a tedious, trial-and-error approach. For this reason, in the last two decades, computational (or theoretical) screening methods have been developed to predict the formation of new cocrystals.^10^ The aim of these computational tools is to reduce the number of experiments by selecting the coformers most likely to yield a cocrystal.

To describe cocrystal formation, two contributions must be taken into account: the miscibility between the API and coformer represented by the short-range intermolecular interactions and the long-range crystal order affected by the molecular packing.^11^ Crystal structure prediction (CSP) considers both contributions to cocrystallization and has demonstrated accurate prediction of cocrystals for small organic molecules.^12^ However, the CSP is based on time-demanding quantum chemical calculations; at the current status, increasing the number of investigated coformers or the molecular size of the API would lead to long-lasting and computationally expensive virtual screening. Therefore, other computational methods, which offer faster screening time and are less resource-intensive, are often employed, despite their reduced accuracy.

Two of these methods are represented by Molecular Complementarity (MC) and COSMOquick. MC was developed by Fábián based on the findings that cocrystallizing molecules tend to have similar shape and polarity.^13^ The approach can be used to rank coformers according to the value of a complementarity score, which describes the similarity of the coformer to the API.^14^ Using a different approach, the COSMOquick software ranks coformer candidates according to their miscibility with the API in a supercooled liquid phase, considering hydrogen bonding, van der Waals forces, and electrostatic interactions.^15–17^ The method is based on the COSMO-RS theory (COnductor-like Screening MOdel for Real Solvents), a thermodynamic liquid-phase theory that combines density functional theory (DFT) calculations and statistical thermodynamics to accurately predict solvation properties of molecules (e.g., solubility).^18,19^ The method is quick in the sense that a fragment-based approach makes use of a large data set of previous DFT calculations, thereby avoiding lengthy computations.^15^ For cocrystal screening, the COSMOquick approach has been retrospectively validated on a set of 318 coformer–API pairs.^17^ MC and COSMOquick have been recently employed for the prediction of cocrystals of API molecules of different sizes.^20,21^

To further streamline cocrystal screening, high-throughput experimental approaches can be employed for the simultaneous and parallel testing of several coformers. Recently, we successfully identified new cocrystals of posaconazole by employing a combined virtual and experimental high-throughput screening (HTS).^22^ Good prediction performances were obtained by the ranking of coformers through the use of the complementarity score. While this prior art shows the promise of these screening methods, there is a need to learn about the performance of a joint approach of using computation and an experimental screening method from prospective case studies in cocrystal research.

In this manuscript, the applicability of the combined approach to discover new cocrystals is tested on the drug cilnidipine (CILP, Figure 1). CILP is a dihydropyridine calcium-channel blocker (reported p*K*_a_ = 11.4)^23^ used in the treatment of hypertension. This drug belongs to BCS class II as demonstrated by its extremely low solubility in water (30–60 ng/mL, BCS class II)^24^ and very high lipophilicity (logP ≈5.5)^23^. CILP was chosen as a model compound because, despite coamorphous systems^25^ and amorphous solid dispersions^26,27^ being reported, no multicomponent crystal forms of the API have been previously discovered. This study aims (1) to find new cocrystals of cilnidipine by using a combined computational and experimental high-throughput screening approach, (2) to evaluate the prediction performance of two computational tools, and (3) to assess the dissolution behavior of the newly discovered cocrystal.

**Figure 1:**
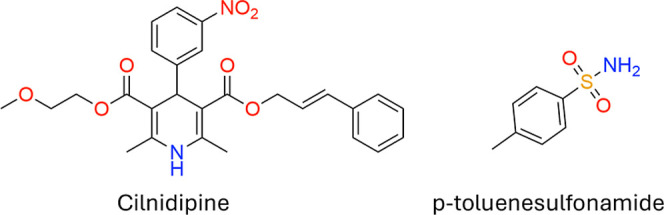
Chemical structures of cilnidipine (CILP) and *p*-toluenesulfonamide (TSA).

## Materials and Methods

### Materials

Cilnidipine (CILP) was purchased from abcr GmbH (Karlsruhe, Germany) and used without further purification. Co-crystal formers were purchased from Merck & Cie (Buchs, Switzerland) and used as received. A list with all the cocrystal formers used in the study is reported in the Supporting Information (Table S1). Sodium hydroxide, sodium dihydrogen phosphate dihydrate, sodium chloride, and acetonitrile (analytical grade) were purchased from Sigma-Aldrich (St. Louis, USA). Trifluoroacetic acid (TFA) was purchased from VWR International (Søborg, Denmark), and Fasted State Simulated Intestinal Fluid (FaSSIF) powder was purchased from biorelevant.com (London, UK).

### Medium Preparations

Blank Fasted-State Simulated Intestinal Fluid (FaSSIF) pH 6.5 (which is a plain phosphate buffer) was prepared by dissolving 0.42 g of sodium hydroxide, 6.18 g of sodium chloride, and 4.47 g of sodium dihydrogen phosphate dihydrate in 950 mL of demineralized water. The pH was adjusted to pH 6.5 with a few drops of a 1 M NaOH solution, and the volume was filled up to 1 L with demineralized water. Standard FaSSIF pH 6.5 was obtained by dissolving 672 mg of FaSSIF powder in 300 mL of blank FaSSIF. The standard FaSSIF differs from the blank version in its inclusion of the biorelevant surfactant lecithin (0.75 mM) and the bile salt sodium taurocholate (3 mM).

### Methods

#### Cocrystal Former Selection

The coformers for the cocrystal screening with cilnidipine were selected after performing a computational screening using COSMOquick on a list of 140 compounds. The top-ranked 52 coformers available in the laboratory were employed in subsequent experimental screening. The 52 coformers were also ranked according to the molecular complementarity (MC) method to compare the prediction abilities of the two computational tools. Details on the two methods are reported in the “Computational Cocrystal Screening” section.

#### High-Throughput Screening

A first “manual” high-throughput screening (HTS-1) for cocrystals was conducted in a 96-well quartz-bottomed microtiter plate with 31 coformers (Table 1). The screening experiments were performed in triplicate; each trial comprised one control well (free drug) and 31 test wells (one for each CILP-coformer pair). First, 0.05 M solutions of cilnidipine and coformers were prepared in different solvents (acetone, methanol, and THF). Then, volumes of solutions containing equimolar amounts of coformer and API were added to the wells according to the plate layout reported in Figure S1. The high-throughput method involved two steps:

- An evaporation step under nitrogen flow;
- A slurry equilibration step conducted by resuspending the solid residues in three different solvents (TBME, methanol, and ethanol) and shaking for several days at 25 °C.

**Table 1:** Summary of Experimental Conditions Employed in the Two High-Throughput Screenings

	HTS 1	HTS 2
no. coformers	31	23
amount API (mg per well)	2.5	2.5
evaporation solvents	THF, MeOH, acetone	ETOH
slurry 1—solvent	ETOH	ETOH
slurry 2—solvent	TBME	acetonitrile
slurry 3—solvent	MeOH	MeOH–H_2_O
detection Type	Raman microscopy	PXRD

At the end of each step, the solid residues were dried under a nitrogen flow and analyzed by Raman microscopy (Figure S3). The plate was covered with aluminum foil during the HTS with the exception of during the Raman microscopy measurements.

A second “semi-automatic” high-throughput screening (HTS-2) was conducted with 23 coformers (Figure S2) in a modified 96-well glass plate, in which the base of the wells can be lifted to the top of the plate thanks to the presence of metal pistons. The screening experiments were performed in quadruplicate. First, 0.05 M solutions of cilnidipine and coformers were prepared in ethanol and loaded onto the Crissy 2002 automated liquid handling platform (Zinsser Analytics, Eschborn, Germany). The screening followed a similar procedure to that of HTS-1 with the robotic system managing all the operations (solvent dispensing, evaporation, and agitation for slurry equilibration). The plate was covered with a pierceable lid during the entire crystallization step to avoid light exposure. Upon completion of the experiments and evaporation of the solvents, the plate was covered with a layer of Kapton foil and the base of the wells was gently raised to press the solid material against the bottom of the Kapton foil. The solid residues were subsequently analyzed by PXRD.

Potential leads, identified by the presence of new features in the Raman spectra or new reflections in the diffraction patterns compared to the reactants, were further investigated by liquid-assisted grinding (LAG) and reaction crystallization^7^ experiments.

### Liquid-Assisted Grinding and Reaction Crystallization Experiments

Liquid-assisted grinding (LAG): CILP (75 mg, 0.152 mmol) and an equimolar amount of coformer were placed into a 3 mL agate milling jar together with 30–50 μL of the solvent (usually ethanol) and two agate milling balls 5 mm in diameter. The mixture was agitated in a Retsch MM200 ball mill (Retsch, Haan, Germany) at the operating frequency of 30 Hz for two 15 min intervals with a cool-down period of 5 min between them. The resultant solid products were tested by FT-Raman spectroscopy.

Reaction crystallization: A solvent in which CILP and the coformer have a similar solubility (at least the same order of magnitude, if possible) was chosen for each experiment (Table S1). CILP (approximately 100 mg) was added either to an almost saturated coformer solution or to an equimolar coformer solution and magnetically stirred for 2 days at RT. Each vial was covered with aluminum foil to prevent light exposure. The suspension was filter-centrifuged through a 0.2 μm PTFE membrane, and the solid phase was characterized by FT-Raman spectroscopy or powder X-ray diffraction (PXRD). In this manuscript, the term “slurry equilibration” refers to HTS experiments, while “reaction crystallization” refers to the reproduced experiment at a larger scale. Despite the difference in scale, both terms describe similar methods.

### Cilnidipine—*p*-Toluenesulfonamide Cocrystal 1:1 (CILP-TSA)

The CILP-TSA cocrystal was scaled up using the reaction crystallization method.^7^ CILP (150 mg, 0.31 mmol) was added to an almost saturated solution (4 mL) of TSA (500 mg, 2.92 mmol, 9.4 equiv) in ethanol and magnetically stirred at RT for 2 days. After a few seconds of stirring, the solution turned yellow. The vial was covered with aluminum foil to prevent light exposure of the suspension. A bright yellow powder was isolated after filtration.

### Solubility Determination

The equilibrium solubility of crystalline CILP was measured in blank FaSSIF at pH 6.5 and in standard FaSSIF at pH 6.5, in the absence and presence of TSA (200 μg/mL, 1.17 mM). Excess crystalline CILP (20 mg, 0.041 mmol) and 10 mL of media were added into screw-cap glass vials. The system was stirred using a magnetic stirring bar at 37 °C. The solubility tests were performed in triplicate. After 24 h, 1 mL of suspension was removed and filtered through Anotop syringe filters (Whatman, Maidstone, UK) with a pore size of 0.2 μm and a 25 mm diameter. The filter was presaturated with 4 mL of a saturated solution of CILP in blank FaSSIF. After discarding the first eight drops, the remaining filtrate was diluted with the mobile phase and quantified by UHPLC-UV.

### Dissolution Studies

Cocrystal dissolution studies were conducted in triplicate in blank FaSSIF (pH 6.5) and FaSSIF pH 6.5. A total of 10.8 mg of the cocrystal (containing 8 mg of CILP and 2.8 mg of TSA; 0.016 mmol) was added to 80 mL of dissolution media in a beaker (height 70 mm, diameter 50 mm). The system was stirred using a magnetic stirrer (1.5 cm, 300 rpm), and the temperature was maintained at 25 °C. Aliquots of 1.0 mL were taken with a syringe at selected time points of up to 24 h and filtered through presaturated Anotop syringe filters (Whatman, Maidstone, UK) with a pore size of 0.2 μm and a 25 mm diameter (the first eight drops were discarded). The filtrate was diluted with mobile phase and analyzed by UHPLC-UV. The final solid residue was recovered through vacuum filtration and was characterized by PXRD. The percentage of cocrystal dissolved was calculated by taking the ratio of the amount of coformer dissolved to the amount of coformer initially added (Table S6).

### Raman Microscopy

The high-throughput plate from HTS-1 was analyzed using a Horiba XploRA PLUS confocal Raman microscope from Horiba Jobin Yvon SAS (Villeneuve d’Ascq, France) equipped with a 100 mW 785 nm diode laser for excitation and a Syncerity OE CCD detector. Measurements were carried out with a long working distance 20× objective, a 50% or 100% laser power, a 1200 lines/mm grating, a 100 μm slit, with 2 accumulations of 30 s each, and a measurement range of 200–2000 cm^–1^.

### FT-Raman Spectroscopy

FT-Raman spectra were recorded on a Bruker MultiRAM FT-Raman spectrometer (Bruker AG, Fällanden, Switzerland) with a near-infrared Nd/YAG laser operating at 1064 nm and a liquid-nitrogen-cooled germanium detector. A total number of 64 scans with a resolution of 2 cm^–1^ was accumulated in the range from 50 to 3500 cm^–1^ using a nominal laser power of 100 mW. The samples were prepared by pressing the isolated powder into an aluminum sample holder.

### Powder X-ray Diffraction

Diffractograms for the high-throughput plate from HTS-2 were collected using a PANalytical X’Pert PRO-MPD diffractometer (Malvern Panalytical Ltd., Malvern, United Kingdom) equipped with a PIXCEL detector operating with Cu K_α,1_ radiation. The measurements were performed in reflection mode using a tube voltage of 45 kV and a current of 40 mA, Soller slit: 0.04, Ni-Filter 0.02 mm, Inc. Mask Fixed 5 mm (MPD/MRD). A step size of 0.013°2θ and a step time of 40.8 s over a 2–34°2θ scanning range were applied. The solid residues of HTS-2 were analyzed by covering the plate with a Kapton foil and by elevating the base of the wells. All sample preparations and measurements were done at RT in an ambient air atmosphere.

Diffractograms of LAG and reaction crystallization samples were collected using a Stoe Stadi P diffractometer (Stoe & Cie. GmbH, Darmstadt, Deutschland) equipped with a Mythen1K detector operating with Cu K_α,1_ radiation. The measurements were performed in transmission mode with a tube voltage of 40 kV and a current of 40 mA. A step size of 0.02°2θ and a step time of 12 s over a 1.5–50.5°2θ scanning range were applied. For a typical sample preparation, about 10–20 mg of the sample was placed between two acetate foils and mounted into a Stoe transmission sample holder. The sample was rotated during the measurement. All sample preparation and measurement were performed at RT in an ambient air atmosphere.

Diffractograms of the residual solids after dissolution experiments were collected by using a Rigaku Miniflex600 diffractometer (Rigaku Corporation, Tokyo, Japan) equipped with a scintillation (NaI) counter detector operating with Cu K_α,1_ radiation. The measurements were performed in reflection mode with a θ/2θ geometry, at a tube voltage of 40 kV, and a current of 40 mA. A step size of 0.02°2θ and a scanning speed of 5°2θ/min over a 3–30°2θ scanning range were applied. For a typical sample preparation, 10–20 mg of powder was added to a sample holder and flattened with the help of a glass slide.

### Thermogravimetry Coupled with Fourier Transform Infrared Spectroscopy

TG-FTIR spectroscopy was performed on a NETZSCH TG 209F1 Libra instrument (Netzsch, Selb, Germany), which was coupled to a Bruker FT-IR Tensor II spectrometer. Around 5 mg of powder was loaded into an aluminum crucible with a (micro) pinhole, and the measurements were carried out under a nitrogen atmosphere and at a heating rate of 10 °C/min over the range of 25–300 °C.

### Proton Nuclear Magnetic Resonance (^1^H NMR)

^1^H NMR analysis was carried out with a Bruker DPX300 spectrometer (Bruker AG, Fällanden, Switzerland) using a proton frequency of 300.13 MHz, a 30° excitation pulse, and a recycle delay of 1 s. Spectra were recorded by accumulation of 16 scans in deuterated DMSO at 298 K. The solvent peak was used for referencing, and the chemical shifts were reported on the tetramethylsilane (TMS) scale.

### Differential Scanning Calorimetry

DSC analyses were carried out with a TA Instruments Q2000 or with a TA Instruments DSC 2500 instrument (TA Instruments, New Castle, Delaware, USA). Approximately 2–3 mg of the sample was heated at a rate of 10 K/min under a nitrogen gas flow (50 mL/min). Standard aluminum sample pans that were hermetically closed were used for the measurements.

### Ultra-high-Performance Liquid Chromatography Coupled with UV–Vis Detection

CILP and TSA were quantified by UHPLC-UV. The UHPLC-UV system (Thermo Fisher Scientific, Massachusetts, USA) consists of an UltiMate Rapid Separation Autosampler (WPS-3000RS), an UltiMate Rapid Separation Thermostated Column Compartment (TCC-3000RS), as well as an UltiMate Rapid Separation UHPLC Diode Array Detector (DAD-3000RS). A Kinetex EVO-C18 column (1.7 μm, 2.1 mm × 100 mm) was used for the separation and kept at a temperature of 40 °C. An isocratic method was employed for the quantification of cilnidipine in dissolution samples in blank FaSSIF. The mobile phase consisted of acetonitrile (70% v/v) and water with 0.1% trifluoroacetic acid (TFA) (30% v/v). Cilnidipine eluted after 2.1 min and was quantified at 240 nm. A calibration curve was built using concentrations from 0.01 to 1 μg/mL. A gradient method was employed for the quantification of cilnidipine in dissolution samples in FaSSIF and TSA. The mobile phase consisted of acetonitrile and water with 0.1% TFA with an elution gradient from 40:60 to 80:20 acetonitrile/water over 1.5 min. An 80:20 ratio was maintained until 4 min and was then changed to 40:60 over 1 min. The system was subsequently equilibrated at a 40:60 acetonitrile/water composition for 5 min. TSA eluted after 1.6 min and CILP after 3.9 min; the compounds were quantified at 224 and 240 nm, respectively. In both methods, a volume of 5 μL was injected and run at a flow rate of 0.3 mL/min.

### Computational Cocrystal Screening

#### Molecular Complementarity

The MC analysis was carried out with the CSD-material package supplied in Mercury 2024.2.0 (Cambridge, UK). The analysis was developed by L. Fábián based on the finding that molecules that cocrystallize tend to have similar shapes and polarities.^13^ To evaluate molecular similarity, 5 molecular descriptors are used: two are related to the polarity of the molecule, namely, fraction of nitrogen and oxygen atoms (FNO) and dipole moment; three are related to the shape and are defined considering the short (*S*), medium (*M*), and long (*L*) axes of a box enclosing the van der Waals molecular volume, namely, the *S* axis, *M*/*L* axis ratio, and *S*/*L* axis ratio (Figure 8). Since the API employed in this study is a large and flexible molecule, the possible conformations in the crystal can vary significantly, leading to different molecular descriptors. To account for these variations, ten likely conformations of the API were generated via the “Conformer generation” function in Mercury 2024.2.0 (Cambridge, UK), starting from the 2D molecular structure of cilnidipine. Generally, one 3D conformation was used for rigid coformers, while three different conformations were generated for flexible coformers. The five molecular descriptors for each conformation of API and coformer were calculated with the CSD-material package supplied in Mercury 2024.2.0 (Cambridge, UK). The molecular descriptors were employed to calculate a normalized complementarity score for each API–coformer pair. As reported by Fábián and Friščić,^14^ the complementarity score is calculated in eq 1 by dividing the difference between the API (*X*_D,API_) and the cocrystal former descriptors (*X*_D,cof_) by the cutoff value (δ_D_) and summing the results for each descriptor (D). The cutoff values were defined by Fábián from a statistical analysis conducted on cocrystals reported in the CSD; the cutoff values for molecular descriptors are defined such that 90% of cocrystals contained in the analysis exhibit a difference in the molecular descriptor that is lower than the cutoff value.^14^ The complementarity score is calculated pairwise for each conformation of cilnidipine and coformer and is then averaged over the 10 conformations of API and the 1–3 conformations of coformer. Different conformations of API and coformers were considered because they might be adopted in the cocrystal. The lower the complementarity score, the larger the similarity between the two molecules and the greater their ability to cocrystallize.

1 $$C_{score}=\sum_{D}\left(\frac{X_{D,API}-X_{D,cof}}{\delta_{D}}\right)=\frac{\left|\Delta M/L\right|}{0.31}+\frac{\left|\Delta S\right|}{3.23}+\frac{|\Delta S/L|}{0.28}+\frac{\left|\Delta dipole\right|}{5.94}+\frac{\left|\Delta FNO\right|}{0.29}$$

Alternatively, the molecular descriptors can be used in the “default settings analysis” available in Mercury (Figure 8). The analysis is usually implemented as a PASS/FAIL test; it predicts cocrystal formation if, and only if, the difference between the API and coformer descriptors is within the cutoff value for all five descriptors. A percentage hit rate is computed for each coformer and is averaged over the number of API conformations screened.

#### COSMO-RS, σ-Surfaces, and σ-Profiles

The COnductor-like Screening Model for Real Solvents (COSMO-RS) is a liquid-phase thermodynamic theory used to accurately calculate thermodynamic properties in solution.^19^ The molecules are placed in a virtual dielectric continuum, and the polarization charge density (σ) on the molecular surface, the so-called σ-surface, is calculated by DFT calculations.^15^ The strength of the theory is the computation of intermolecular interactions as local contacts on the σ-surface by statistical thermodynamic calculations of pairwise interactions of surface segments;^18,19^ hydrogen bonding, van der Waals, and electrostatic interactions are accurately evaluated in this way. For computation, the σ-surface is represented by a histogram of segmental screening charge densities, which is called the σ-profile. The calculation of intermolecular interactions leads to the determination of the energy and the chemical potential of the system, from which thermodynamic properties can be derived.^28^

#### COSMOquick Screening

Since COSMO-RS is a liquid-phase thermodynamic theory, it is assumed that the interactions in a crystal are similar to those in a supercooled liquid. The strength of interactions between two components can be estimated by the excess or mixing enthalpy (Δ*H*_mix_) of an API–coformer pair under supercooled conditions compared to the pure components.^16,17^ The mixing enthalpy is a rough approximation of the free energy of cocrystal formation (Δ*G*_cc_), as presented in eq 2.

2 $${\Delta G}_{cc}={\Delta H}_{mix}-{T\Delta S}_{mix}-{\Delta \Delta G}_{fus}\approx {\Delta H}_{mix}$$

The difference between the energy of fusion of the cocrystal and reactants, ΔΔ*G*_fus_, representing the long-range and packing contribution to cocrystallization, is assumed to be negligible by this approach, and at supercooled conditions the entropy term is assumed to be negligible. The tendency of an API–coformer pair to form a cocrystal is evaluated by the screening function *F*_screen_. This function accounts not only for the molecular interactions (Δ*H*_mix_) but also for the flexibility of the molecule in the form of the number of rotatable bonds.^16,17^ The screening function is reported in eq 3, where *a* is a fit parameter to be determined on a set of experimental results, and *n* is the total number of rotatable bonds of the drug and the coformer. *F*_screen_ is related to the tendency of cocrystal formation because it accounts for the mixing enthalpy (Δ*H*_mix_) of a coformer-API pair but, in addition, penalizes floppy molecules, as it was observed experimentally that they hinder cocrystal formation.^16,17^ The lower the *F*_screen_ value for a coformer-API pair, the higher the likelihood of cocrystal formation.

3 $$F_{screen}={\Delta H}_{mix}+a\left(\max\left(1,n_{drug}\right)+\max\left(1,n_{coformer}\right)\right)$$

COSMOquick speeds up the calculation of σ-surfaces by combining surface fragments from a database of precomputed molecules. The database contains precomputed σ-surfaces at the BP-TZVP-COSMO level.^29–31^ The virtual cocrystal screening of cilnidipine was performed using the COSMOquick software v.2020 (Dassault Systèmes/Biovia) on a list of 140 compounds by selecting a 1:1 CILP/coformer stoichiometry. The 1:1 ratio was selected for the screening because it is the most common cocrystal stoichiometry. A list of Simplified Molecular Input Line-Entries (SMILES) of CILP and the coformers was employed as input for the calculation. Most of the coformers’ σ-profiles were available in the compound database, while approximate σ-profiles were generated for CILP by COSMOfrag. The statistical thermodynamic calculations were carried out by using the COSMOtherm code. The output resulted in a list of coformers ranked according to the values of the penalty function (*F*_screen_) or the excess enthalpy (Δ*H*_mix_) of the corresponding cocrystals with cilnidipine.

## Results and Discussion

### Experimental Cocrystal Screening

The CILP-TSA cocrystal was discovered both by “manual” and “semi-automatic” high-throughput screening methods (Figure 2). The starting CILP powder was an anhydrous racemate, as its diffraction pattern matched that calculated from the previously determined crystal structure [CCDC reference 2215828].^32^ Although a second polymorph of the compound has been reported,^33^ it was not observed in this study. In addition, since photodegradation of cilnidipine was described in concentrated solutions in methanol,^34^ precautions were taken to prevent light exposure during crystallization experiments.

**Figure 2:**
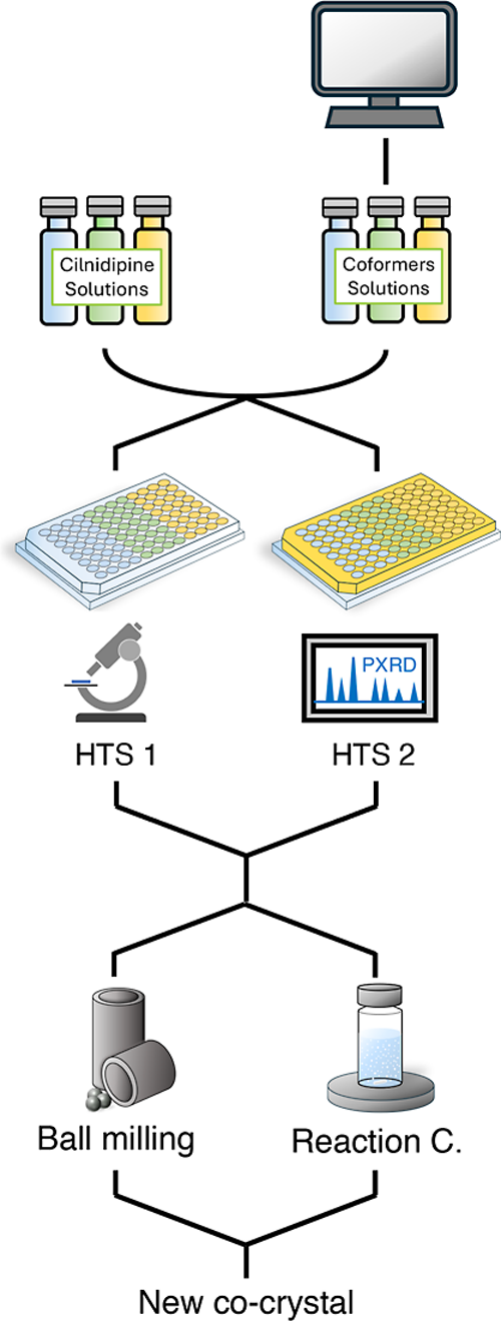
Schematic representation of the two high-throughput screening approaches (HTS-1 and HTS-2) employed in this study. The term “Reaction c.” is an abbreviation of “reaction crystallization”.

Coformers were selected through a prospective computational approach performed with the COSMOquick method: the top-ranked 52 coformers available in the laboratory were employed in the HTS (Table S1). Coformers were tested experimentally by solution-based methods such as evaporation and slurry equilibration. To increase the likelihood of cocrystallization, replicates were performed in different solvents (Table 1 and Figures S1, S2). Indeed, cocrystal stability and formation strongly depend on the relative solubility of the API and coformer in the solvent used.^35^

The first approach (HTS-1) involved manual operations for the dispersion and addition of solvents and the detection of crystals. Indeed, new solid phases were identified by looking for crystals in the wells and collecting Raman spectra using a Raman microscope. The presence of new features in the Raman spectrum indicates the formation of new interactions in the solid phase. A strong lead for a potential cocrystal was obtained with *p*-toluenesulfonamide (TSA) because both crystals with new morphology (Figure S3) and a new Raman spectrum (Figure 3a) were observed in multiple wells: i.e., evaporation experiments with THF and slurry equilibration with *tert*-butyl methyl ether (TBME), ethanol (EtOH), and methanol (MeOH). Only weak leads were obtained with a few of the other coformers (Figure S1 and Table S1) because only one or two additional low-intensity peaks were observed in a single solvent.

**Figure 3:**
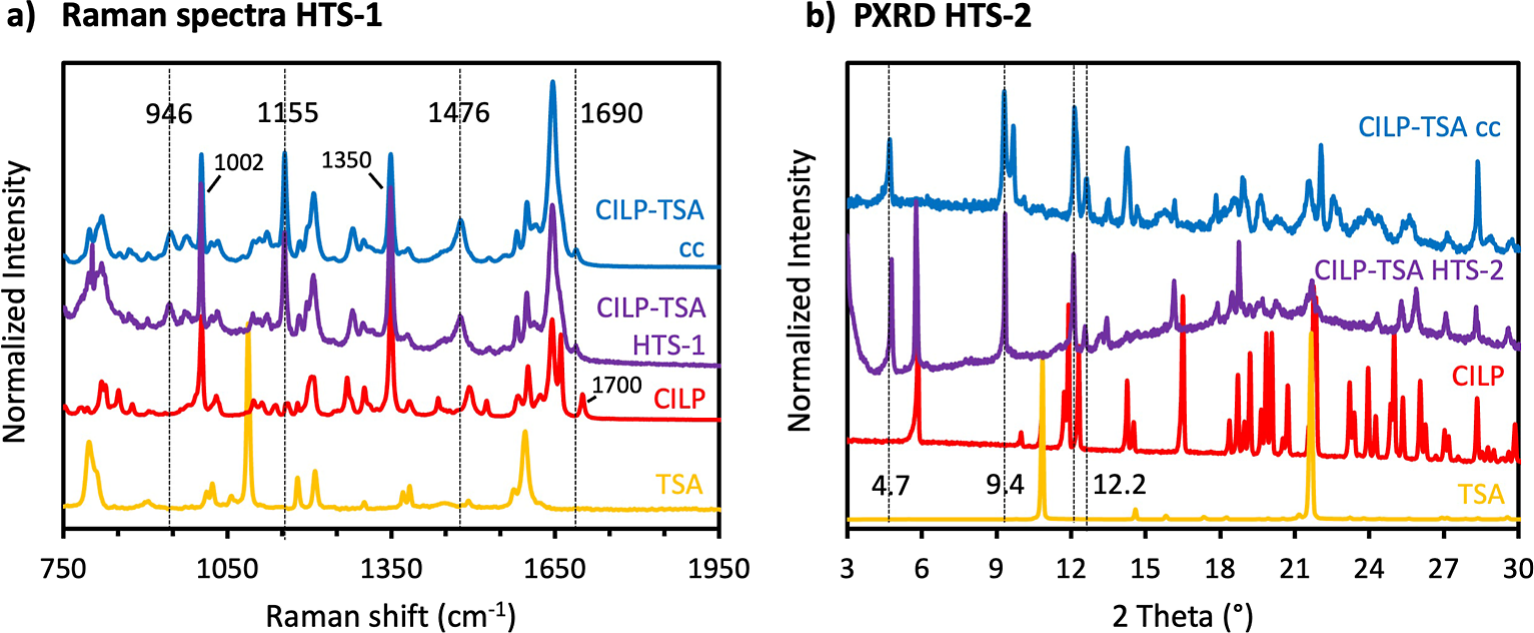
(a) Overlay of Raman spectra and (b) overlay of diffraction patterns. From the bottom, *p*-toluenesulfonamide (yellow), cilnidipine (red), CILP-TSA HTS-1 experiment—well G4 (purple-Raman), CILP-TSA HTS-2 experiment—well F1 (purple-PXRD), and CILP-TSA cocrystal (blue).

Based on these observations, a second HTS approach (HTS-2) was conducted on 21 new coformers with the addition of TSA and naphthalene-2-sulfonamide as positive and negative controls, respectively, in order to confirm the HTS-1 results. The HTS-2 approach involves automatic operations performed by a robotic system (e.g., dispensing of solution, evaporation, and shaking) and allows the analysis of the solid residues directly by PXRD. Similarly to HTS-1, strong leads of HTS-2 were observed only with TSA. A new diffraction pattern, overlapping with low-angle CILP reflections, was observed in multiple wells containing the coformer TSA (Figures 3b and S2), indicating the presence of a new crystal phase. Weak leads were observed with amino acids, such as glycine, l-valine, and l-alanine. These results confirmed that using a semiautomatic HTS approach leads to the same identification of TSA as the manual one with the advantage of an accelerated screening time.

It is worth noting that some crystallization experiments, both in HTS-1 and HTS-2, resulted in the formation of a transparent and yellowish amorphous material, especially by evaporation from acetone and THF. The precipitation of the amorphous phase might be due to the nature of cilnidipine, which is reported to be a slow crystallizer from the melt^36,37^ or due to fast evaporation conditions. Furthermore, the presence of the coformer may result in the formation of a coamorphous system with high kinetic stability rather than a cocrystal.^25^

Since cocrystal formation may occur only with specific crystallization methods and conditions, the HTS findings with TSA and other weak lead coformers were reproduced at the 100 mg scale by liquid-assisted grinding (LAG) and reaction crystallization experiments (Table S1). LAG is a mechanochemical method that has previously proven to be successful in occasions in which the solvent-based methods failed.^8^ In addition, reaction crystallization experiments at RT were performed by using a nearly saturated solution of the coformer to facilitate access to the stability region of the cocrystal.^7,35^ A new crystal phase was observed only with TSA using three different crystallization methods (LAG, reaction crystallization, and evaporation) and two different solvents (i.e., ethanol and acetonitrile), while physical mixtures were obtained with the other coformers.

The newly discovered phase is a 1:1 CILP—TSA cocrystal (see the “Co-crystal Characterization” section). The characteristic Raman spectrum and diffraction pattern of the cocrystal perfectly overlap with the features observed in the HTS experiments, as demonstrated by the Raman peaks at 1155, 1476, and 1690 cm^–1^ and by the powder X-ray reflections at 4.7, 9.4, and 12.2°2θ (Figure 3), further corroborating the high-throughput screening results.

These findings indicate that new cocrystals of an API can also be identified using a more automated HTS approach (HTS-2) in addition to the standard manual approach (HTS-1). The advantages of the semiautomated HTS-2 approach are the reduced user operation and faster analysis time of the entire well with PXRD. On the other hand, the manual HTS-1 approach allows for a more accurate investigation of the individual crystalline materials in the wells and for identification of complexation as amorphous materials using Raman microscopy.

### Cocrystal Characterization

The discovered multicomponent crystal of CILP with *p*-toluenesulfonamide (CILP-TSA) was obtained through the reaction crystallization method in ethanol. Due to the neutral nature of both CILP and TSA, the multicomponent crystal must be a cocrystal rather than a salt. The crystals of CILP-TSA display a bright yellow color compared with the pale-yellow crystalline powder of CILP (Figure 4a,b). The cocrystal is characterized by a 1:1 stoichiometry of the components, as determined by ^1^H NMR spectroscopy and UHPLC-UV (Figure S7 and Table S5). The DSC thermogram of CILP-TSA shows one endothermic event at 122.6 °C corresponding to the cocrystal melting point (Figure 4c), which is located in between the ones of CILP (105.8 °C) and TSA (137.7 °C).^38^ TG-FTIR analysis revealed that the cocrystal is anhydrous; no mass loss was detected before the degradation of the components at 250 °C (Figure S6). The presence of new features in the Raman spectrum of the CILP-TSA cocrystal (Figure 3a) indicates the formation of intermolecular interactions, such as hydrogen bonds, between the API and coformer in the solid state. The Raman spectrum of crystalline CILP (red in Figure 3a) is characterized by the C=O carbonyl stretching vibration of the ester group at 1700 cm^–1^,^23^ the C=C stretching vibrations of the aromatic rings, the conjugated double bond and the dihydropyridine ring (1661, 1644, and 1600 cm^–1^), the symmetrical stretching of the NO_2_ group at 1350 cm^–1^,^39,40^ and the=C–H in-plane deformation vibrations of the aromatic rings at 1002 cm^–1^ and 1030 cm^–1^.^23,41^ In addition to the features of CILP, the Raman spectrum of the cocrystals (blue in Figure 3a) shows a shift in the C=O carbonyl stretching frequency of the ester group (1690 cm^–1^), a new broad band at 946 cm^–1^, which may be associated with C–O–C stretching vibration of the aliphatic ether group, and the S=O symmetric stretching of the sulfonamide moiety of TSA at 1155 cm^–1^.^41,42^ These features suggest the formation of intermolecular interactions in the solid state between the ester and ether groups of CILP and the sulfonamide group of TSA.

**Figure 4:**
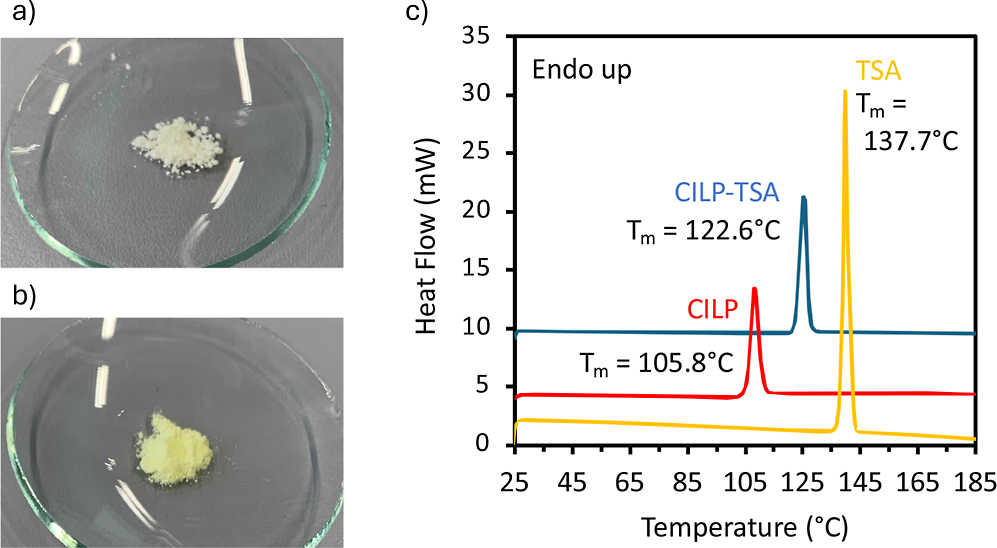
(a) Picture presenting the pale-yellow powder of crystalline CILP; (b) picture presenting the bright-yellow crystalline powder of the CILP-TSA cocrystal; (c) DSC thermograms of crystalline CILP, TSA, and the CILP-TSA cocrystal (enthalpy of fusion is reported in the Supporting Information, Table S4).

Figure 5 (left-hand side) presents the cocrystal dissolution profiles in blank FaSSIF pH 6.5 and FaSSIF pH 6.5. The standard FaSSIF medium differs from blank FaSSIF, a plain phosphate buffer, by the presence of the biorelevant surfactant lecithin and the bile salt sodium taurocholate. The cocrystal achieves maximum concentrations in solution that are 6.5 times (blank FaSSIF) and 4 times (FaSSIF) above the solubility of the crystalline drug. After maximum supersaturation is achieved—which is after 4 h in blank FaSSIF and 3 h in FaSSIF—a slow decrease in concentration corresponding to precipitation of the crystalline drug is observed. Supersaturation is still sustained after 8 h from the beginning of the dissolution experiment. The dissolution profile in FaSSIF pH 6.5 mimics how the cocrystal would dissolve in the small intestine after administration with a glass of water. Higher drug solubility is reported in this medium due to the presence of amphiphilic molecules (sodium taurocholate and lecithin), which form mixed micelles that are able to solubilize CILP (Table 2).

**Figure 5:**
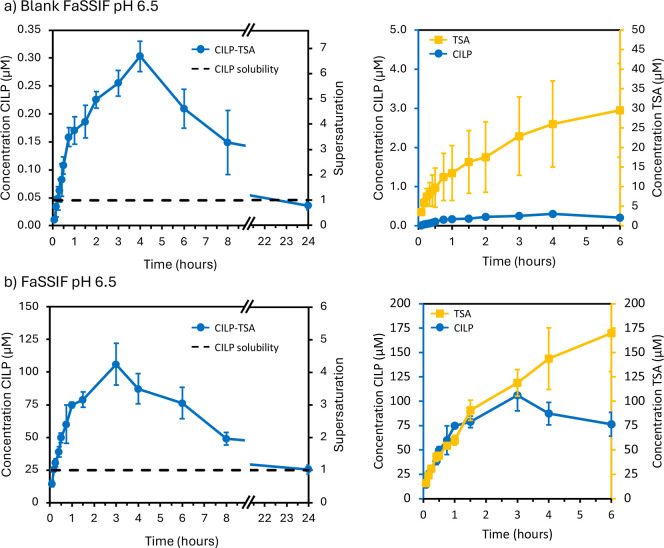
(a) Left: co-crystal dissolution profile in blank FaSSIF pH 6.5; right: comparison of CILP and TSA bulk concentrations during cocrystal dissolution (note the different scales and units of the vertical axes); (b) left: cocrystal dissolution profile in FaSSIF pH 6.5; right: comparison of CILP and TSA bulk concentrations during cocrystal dissolution. Data are plotted as mean value (*n* = 3) ± standard deviation, which is represented by the error bars. The dashed black line represents the solubility of crystalline cilnidipine in the dissolution medium. No change in pH in the media was measured after 24 h.

**Table 2:** Solubility of Cilnidipine in Blank FaSSIF and FaSSIF pH 6.5 in the Presence and Absence of the Dissolved Coformer at 37 °C^a^

	solubility blank FaSSIF (μM)	solubility FaSSIF (μM)
CILP	(4.6 ± 0.6) × 10^–2^	25.0 ± 3.9
CILP (+200 μg/mL TSA)	(5.0 ± 0.4) × 10^–2^	29.9 ± 3.0

a The tested coformer concentration (200 μg/mL, 1.16 mM) is five times higher than the maximum concentration achievable during the dissolution studies (43 μg/mL, 0.25 mM). Values are presented as mean (*n* = 3) ± standard deviation. The final measured pH was 6.5.

The supersaturation achieved through cocrystal dissolution in FaSSIF results in potentially increased drug absorption in vivo compared to the crystalline drug.

Table 2 reports no significant difference (*p* > 0.05) between the drug solubility values measured in the absence and presence of coformer, confirming that no interactions between CILP and TSA are present in solution. Since no complexation with the coformer takes place in solution, the drug supersaturation achieved by this cocrystal is purely dependent on the properties of the multicomponent crystal.

Insights into the cocrystal dissolution–supersaturation–precipitation (DSP) behavior can be gained by comparing CILP and TSA bulk concentrations during dissolution as reported on the right side of Figure 5a (blank buffer) and Figure 5b (FaSSIF). Theoretically, due to the nature of the crystal phase, cocrystals dissolve congruently according to their stoichiometry. For instance, the CILP-TSA cocrystal, characterized by a 1:1 stoichiometry of the components, would release one millimole of CILP for every millimole of TSA into the dissolution medium. However, Figure 5a shows that, already after 5 min of dissolution in blank FaSSIF, the bulk molar concentration of TSA (3.05 μM) is more than 100 times higher than that of CILP (0.01 μM). This behavior may be explained by a rapid cocrystal dissolution that generates high supersaturation adjacent to the surface of the cocrystal particles, while the bulk concentration is still below the drug solubility. The supersaturation on the surface of the cocrystal is the driving force for heterogeneous nucleation of CILP crystals.^43^ Examples of surface-nucleating cocrystals have been reported in the case of carbamazepine (CBZ), where needle-like crystals of CBZ grew on the surface of several cocrystals of the drug.^44,45^ The process can be regarded as a particle surface-solution-mediated phase transformation (PS-SMPT).

Despite the nucleation of crystalline cilnidipine, the bulk CILP concentration continued to increase, reaching a critical value of 0.3 μM after 4 h, corresponding to a supersaturation of 6.5. This maximum concentration marks the critical supersaturation at which the bulk growth of cilnidipine crystals occurs, and precipitation begins to outcompete cocrystal dissolution.

Figure 5b shows a different dissolution behavior of the cocrystal in FaSSIF. A stoichiometric ratio of CILP to TSA is observed in the bulk solution up to nearly 3 h, when the maximum supersaturation is reached. These results suggest sustained congruent dissolution over a prolonged time. Nucleation and growth of CILP crystals occur in the bulk solution, triggered by reaching the critical supersaturation value. The different cocrystal dissolution behavior observed in blank FaSSIF and FaSSIF can be explained by the presence of surfactant micelles in FaSSIF. Lipert et al. showed that surfactant micelles reduce the achievable cocrystal solubility advantage (ratio of cocrystal to drug solubility) and, hence, the driving force for precipitation.^46^ The presence of micelles, able to solubilize the drug molecules, may decrease the rate of supersaturation, preventing fast nucleation of crystalline CILP around the cocrystal surface. Bile salts and lecithin, present in FaSSIF, may also act as precipitation inhibitors for crystalline CILP.

In addition, the faster dissolution of the cocrystal in FaSSIF as compared to blank buffer, evaluated by the dissolved concentration of TSA, may be explained by better wettability of the crystals and higher affinity of CILP for the medium: the solubility of CILP in FaSSIF is 600 times the one observed in blank FaSSIF (Table 2).

The final solid residues collected at the end of the experiment confirmed the reprecipitation of crystalline CILP (Figure 6). Poor wettability of the cocrystal powder and agglomeration during the dissolution in blank FaSSIF resulted in only a third of the cocrystal dissolving after 24 h (Table S6). Figure 6 shows that most of the solid residue collected after dissolution in blank FaSSIF belongs to the cocrystal. The better powder wettability achieved in FaSSIF and the lack of rapid heterogeneous nucleation on the crystal surface resulted in an almost complete dissolution of the cocrystal (Table S6).

**Figure 6:**
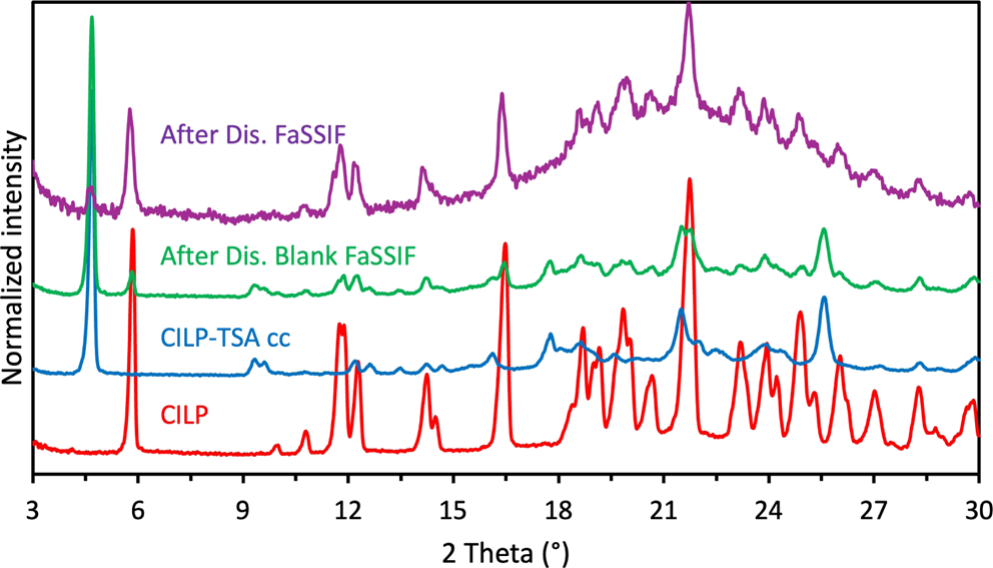
Overlay of diffraction patterns of the solid residues collected after dissolution studies and CILP-TSA and CILP. From the bottom: cilnidipine (red), CILP-TSA (blue), solid residue after dissolution in blank FaSSIF (green), and solid residue after dissolution in FaSSIF (purple).

#### Computational Cocrystal Screening

Computational tools can be employed prior to the experimental cocrystal screening to rank the most promising coformers from a library of compounds. The purpose of this approach is to reduce the number of experiments and increase the efficiency of cocrystal formation by testing the top-ranked coformers first. In this study, two computational methods, Molecular Complementarity (MC) and COSMOquick, were employed to rank coformers in the cocrystal screening of cilnidipine. The prediction performance of the two methods was then evaluated based on the experimental findings.

MC revealed an excellent prediction ability in selecting the best coformers for CILP, because TSA, the only coformer resulting in a cocrystal to date, was ranked third out of 52 compounds (Tables S1 and S2). In contrast, COSMOquick ranked TSA in the middle of the screening list (22nd out of 52). Since TSA is the third-ranked coformer, the use of MC to preselect most likely coformers would have reduced the number of experiments required to discover a cocrystal. Cocrystal formation depends on both the intermolecular interactions between the components and the packing contributions to the long-range order. Therefore, the difference in prediction performance of the two computational methods may be related to the approach used: MC considers the similarity in shape and polarity of the API–coformer pairs, while COSMOquick considers the van der Waals and electrostatic interactions between them.

The worse performance achieved with COSMOquick might be explained by two factors: (1) the approximations included in the method and (2) the incorrect representation of the cilnidipine molecule by the COSMOquick fragmentation approach.

COSMOquick assumes that the interactions in the cocrystals are similar to a supercooled liquid phase.^16^ For this reason, the approach ranks coformers according to their “miscibility” with cilnidipine (Δ*H*_mix_), namely, the strength of interactions with the API compared to those of the two pure components in the supercooled liquid. The method neglects the contributions of crystal packing and long-range order to cocrystal formation (ΔΔ*G*_fus_ term in eq 2) that may prevent the formation of some interactions. Despite the approximation, the method was successfully tested on different cocrystal cases.^16,17^ Nevertheless, crystal packing contributions may be more relevant for large and flexible molecules and, hence, cannot be neglected in the prediction of cocrystals. This is possibly the case for cilnidipine, where the approximations lead to an incorrect ranking of the TSA.

An additional reason for the incorrect ranking could be due to the σ-surface of cilnidipine. The σ-surface represents the polarization charge density surface and is employed in the calculation of intermolecular interactions (Figure 7): the more imprecise the σ-surface representation, the less accurate the estimation of the intermolecular interactions will be. In COSMO-RS, the σ-surfaces are obtained through DFT calculations, but COSMOquick, in order to reduce computational time, generates them by combining one or more precomputed fragments from a database.

**Figure 7:**
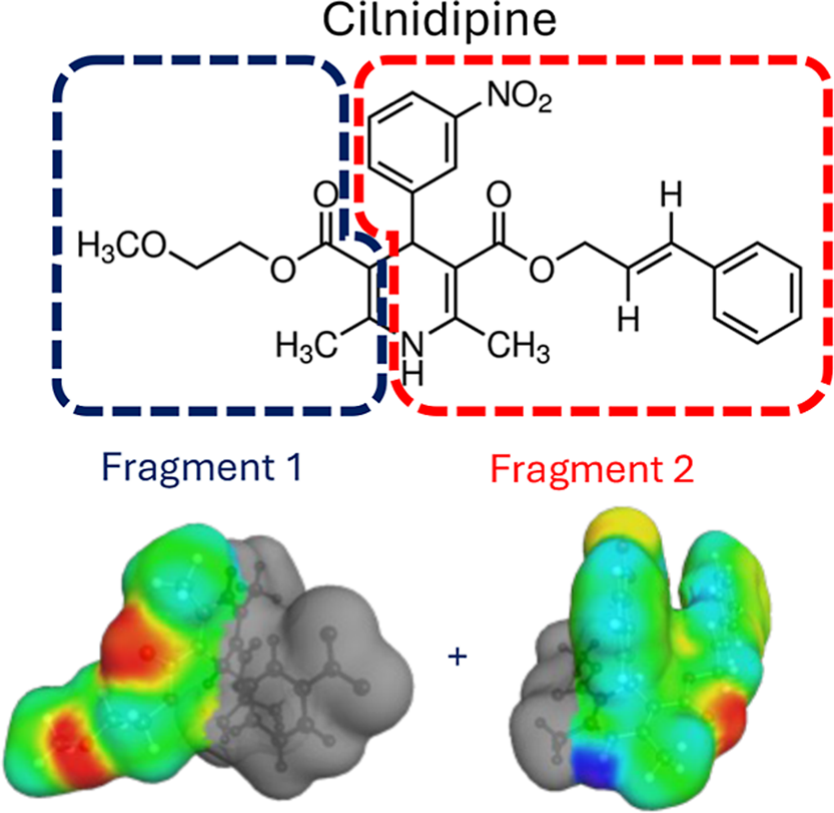
COSMOquick precomputed fragments employed to build the σ-surface of cilnidipine. Areas with positive polarization charge density are highlighted in red (H-bond acceptor), while areas with negative polarization charge density are highlighted in blue (H-bond donor).

The σ-surfaces of the coformers employed in the study were already present in the COSMOquick database, but the σ-surface of cilnidipine was obtained by combining two different fragments, which cut the dihydropyridine ring in two parts (Figure 7). The fragmentation may lead to an incorrect representation of the polarization charge density of the dihydropyridine ring and of the hydrogen bond donor (N–H). This could result in an erroneous estimation of interactions with the coformers. The importance of the N–H group is shown by the crystal structures of cilnidipine and other dihydropyridines (e.g., nifedipine [CCDC reference 2263411]), where monodimensional N–H–O hydrogen-bonded chains dominate the crystal packing.^32^ In addition, the σ-surface of the COSMOquick approach does not account for the different possible conformations of cilnidipine, which can be otherwise considered in the more laborious COSMOtherm calculations.

Therefore, the use of COSMOquick as a screening tool for promising coformers is suggested when the σ-surface of the API is not subjected to fragmentation but has been previously calculated at a quantum chemical level or when the fragmentation occurs far from H-bond donors or acceptors.

It is worth mentioning that the COSMOquick ranking may be used as a screening method for the prediction of coamorphous systems. Indeed, differently from the crystal phase, packing contributions and solid-state order are negligible in the amorphous phase. In the experimental HTS of CILP, an amorphous material was observed in the presence of some coformers. However, testing such an approach would be beyond the scope of this paper.

On the contrary, packing contributions might be caught by MC through the calculation of the complementarity score (*C*_score_). Indeed, coformers are ranked according to the *C*_score_ values, which employ five molecular descriptors—three related to the shape and two related to the polarity—to describe the similarity between API and coformers (Figure 8). A low *C*_score_ would indicate that the molecules have similar shapes, and it will be easier to find a close packing of the API and coformer in the same multicomponent crystal. In addition, different conformations of the API and coformers are included in the model because they can be adopted in the cocrystal. A multicomponent score is calculated for every pair of API conformation–coformer conformation; then the final score is averaged over the total number of pairs (usually 10 API conformations). The MC method was suggested to work better when dealing with APIs where hydrogen bonding is expected to be weaker, such as for artemisinin.^47^ However, the case of CILP looks different; the molecule possesses hydrogen bond donor and acceptor groups, and strong interaction might be established, but differently from COSMOquick, the MC analysis might be able to account for adverse packing with the coformer, which prevents the formation of synthons in the solid state.

**Figure 8:**
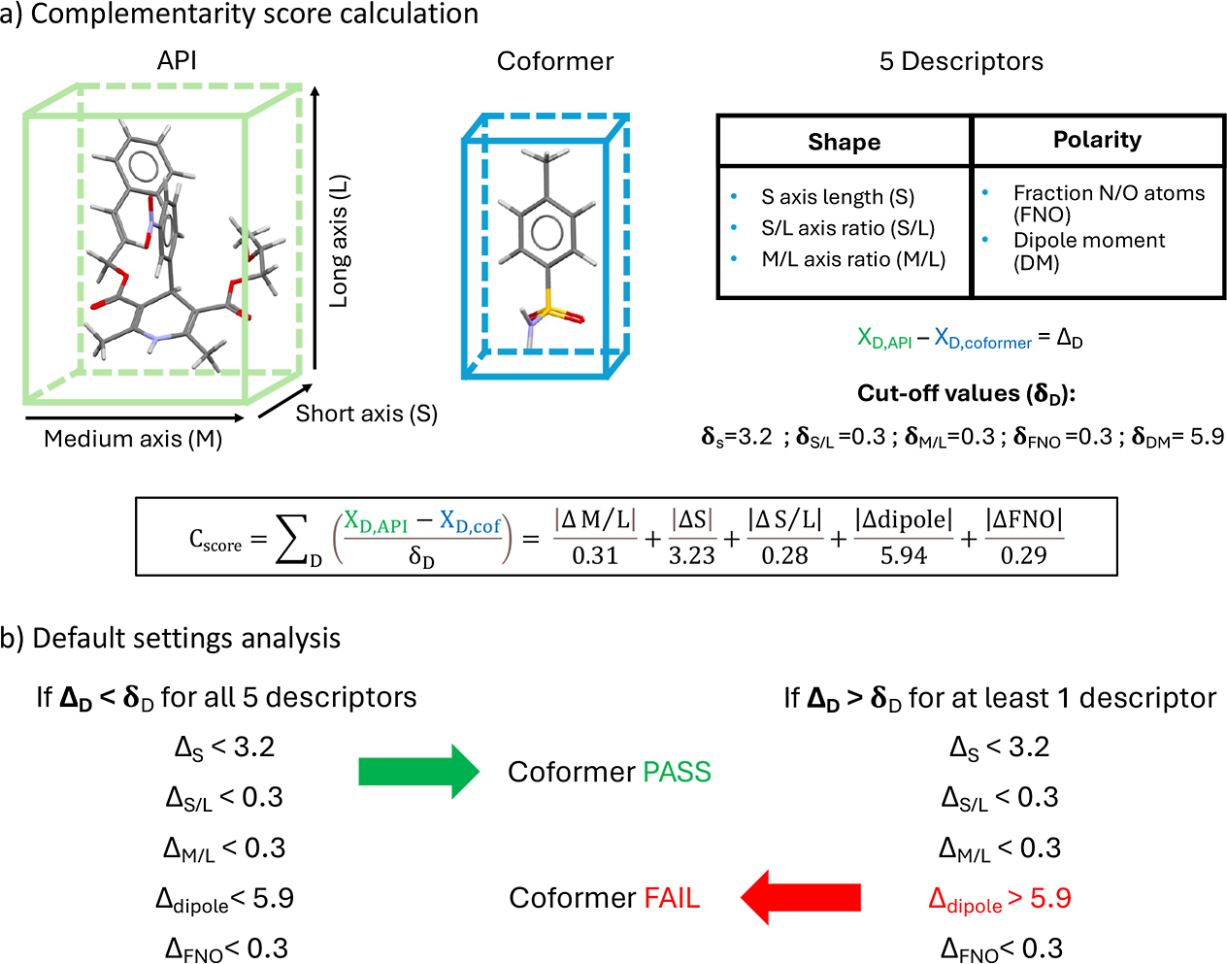
(a) Schematic representation of the MC approach used to calculate the complementarity score (*C*_score_) of CILP and coformers.^14^ Molecular descriptors representing the shape and polarity of the molecules are employed in the calculation. Cut-off values were determined by Fábián based on a statistical analysis performed on cocrystals contained in the CSD.^13,14^ (b) Schematic representation of the passing rule used in the default settings of the MC cocrystal screening available in Mercury.

An alternative screening approach performed with the MC method can be conducted by using the default settings provided in Mercury (Figure 8). In this case, the ranking with complementarity scores is substituted by a strict discrimination of coformers that form and do not form cocrystals. Cocrystal formation is predicted if, and only if, the difference of API and coformer descriptors is within the cutoff value for all five descriptors. The coformers tested with cilnidipine failed this test with every API conformation, resulting in a “FAIL” classification for all coformers (Table S3). Therefore, the MC analysis conducted with Mercury default settings missed the identification of the cocrystal of CILP with TSA, unlike the *C*_score_ screening. Researchers at the CCDC have recently performed a validation exercise on the MC approach with the default Mercury settings using a larger data set of cocrystals (2500 cocrystals employed) consisting of both positive and negative observations.^48^ The validation showed that MC has variable accuracy with small drug molecules and suggested the use of the computational methods only on compounds with similar features to the ones contained in Fábián’s data set (small, neutral molecules with MW < 300 Da).

The good prediction performance obtained with CILP (MW = 492.5 Da) and posaconazole (Da = 700.8 Da)^22^ contrasts with these findings and suggests that ranking coformers using a screening function (*C*_score_) is more effective for identifying new cocrystals than relying on a strict cutoff value discrimination (MC with default settings). Indeed, the purpose of the computational approach is to reduce the number of cocrystallization experiments by prioritizing coformers at the top of the ranking list, without needing to define a cutoff value to accurately predict every coformer tested. Such an approach may be beneficial in the pharmaceutical industry, where discovered molecules are becoming larger and larger, and it is sufficient to increase the efficiency of experimental cocrystal formation by focusing on the top-ranked coformers.

Many prospective cocrystal screenings in the literature have tested the MC method with the default settings method, but only a few have calculated the complementarity score. Testing the applicability of the complementarity score on a larger number of cocrystal systems could further confirm the prediction performance of the method.

## Conclusions

A new cocrystal of cilnidipine (CILP) with *p*-toluenesulfonamide (TSA) characterized by a 1:1 stoichiometry of the components was discovered in this study. The CILP-TSA cocrystal was identified both by manual and semiautomatic experimental high-throughput screening approaches from a subset of 52 coformers tested, allowing for a rapid and successful test of several coformer candidates. In addition, the computational tool Molecular Complementarity (MC) demonstrated good prediction performance for the CILP cocrystal by ranking TSA as the third favorite candidate in the list of 52 coformers, based on the complementarity score. The MC tool proved to be valuable in the preselection of promising coformers and in the potential reduction of experimental trials. Prediction outcomes suggest that crystal packing contributions for cocrystal formation need to be considered when dealing with large and flexible APIs.

Characterization of the CILP-TSA cocrystal revealed sustained supersaturation over 8 h in dissolution studies conducted in blank FaSSIF and FaSSIF pH 6.5. These results suggest that the cocrystal can achieve higher exposure and favor drug absorption compared to crystalline CILP. The combination of computational methods and high-throughput experimental screening can allow for faster discovery of cocrystals of newly developed chemical entities in the pharmaceutical industry.

## Supplementary Material



## Notes

List of coformers employed in the cocrystal screening; ranking of coformers based on COSMOquick and MC methods and comparison with HTS results; HTS experimental details; solution ^1^H NMR spectra and thermogravimetry for the CILP starting material and the new cocrystal obtained; enthalpy of fusion of API, coformer, and cocrystal; and percentage of dissolved cocrystals (PDF)

## Abbreviations

API: active pharmaceutical ingredient
BCS: Biopharmaceutics Classification System
CBZ: carbamazepine
CILP: cilnidipine
CCDC: Cambridge Crystallographic Data Center
COSMO-RS: COnductor-like Screening MOdel for Real Solvents
CSD: Cambridge Structural Database
CSP: crystal structure prediction
DFT: density functional theory
DSC: differential scanning calorimetry
FaSSIF: fasted state simulated intestinal fluid
FNO: fraction of nitrogen and oxygen atoms
^1^H NMR: proton NMR
HTS: high-throughput screening
LAG: liquid-assisted grinding
MC: molecular complementarity
PS-SMPT: particle surface-solution-mediated phase transformation
PXRD: powder X-ray diffraction
RC: reaction crystallization
TSA: *p*-toluenesulfonamide
UHPLC-UV: ultra-high-performance liquid chromatography coupled with UV detection.
