# Supplementary material for: Discovery of Cilnidipine Cocrystals with Enhanced Dissolution by the Use of Computational Tools and Semiautomatic High-Throughput Screening
Source: Cryst Growth Des. 2025 Apr 29;25(10):3374–85. doi: 10.1021/acs.cgd.5c00184 (PMC12100653; doi:10.1021/acs.cgd.5c00184)
Supplement: Supplementary file 1 [file cg5c00184_si_001.pdf]

Discovery of cilnidipine co-crystal with enhanced dissolution by the use of computational tools and semiautomatic high-throughput screening

Supporting Information

Matteo Guidetti [a,b], *Rolf Hilfiker* [a], *Susan M. De Paul* [a], *Annette Bauer-Brandl* [b], *Fritz Blatter* [a],

Martin Kuentz [c]

Corresponding author: Martin Kuentz

[a]Solvias AG, Solid-State Development Department, Römerpark 2, CH- 4303 Kaiseraugst, Switzerland

[b]University of Southern Denmark, Department of Physics, Chemistry and Pharmacy, Campusvej 55, 5230 Odense, Denmark

[c]University of Applied Sciences and Arts Northwestern Switzerland, Institute of Pharma Technology, CH- 4132 Muttenz, Switzerland

1 CO-CRYSTAL SCREENING 3

1.1 List of Coformers with Summary of Computational and Experimental Results 3

1.2 Molecular complementarity results: complementarity score 8

1.3 Molecular complementarity results: hit rate calculation using default Mercury settings 10

2 CILNIDIPINE (CILP) 13

2.1 TG-FTIR 13

2.2 Proton Nuclear Magnetic Resonance (^{1}H-NMR) 14

3 CILNIDIPINE – *P*-TOLUENESULFONAMIDE 1:1 CO-CRYSTAL (CILP-TSA) 15

3.1 TG-FTIR 15

3.2 Proton Nuclear Magnetic Resonance (^{1}H-NMR) 16

3.3 Melting point and enthalpy of fusion co-crystal 17

3.4 UHPLC-UV Co-crystal 17

4 CO-CRYSTAL DISSOLUTION STUDIES 17

1 Co-crystal Screening

1.1 List of Coformers with Summary of Computational and Experimental Results

Table S1. List of the 52 coformers employed in the co-crystal screening of cilnidipine. The coformers are ranked according to the s the F_{screen} values computed by COSMOquick. For each coformer the following parameters are reported: the abbreviated name, the F_{screen} and Cscore values, the type of HTS performed, the experimental results (red = fail; yellow = weak leads with new Raman peaks or PXRD reflections observed; blue = strong leads with an entirely new Raman spectrum or diffraction pattern observed), and in some cases, the solvents used.

Coformer name	Abbrev.	Computational screening					Experimental results				
		ΔH_{mix} (COSMOquick)	F_{screen} (COSMOquick)	Rank (COSMO)	C_{score} (MC)	Rank (MC)	HTS Type	HTS	LAG	Solvent evaporation	Solvent evaporation
Naphthalene-1,5-disulfonic acid	NDS	-3.86	4.30	1	3.60	16	1		ETOH		
Oxalic acid	OXA	-2.64	5.00	2	5.18	52	1		MeCN	1-butanol	
Benzensulfonic acid	BES	-1.91	5.23	3	2.75	4	1				
p-toluenesulfonic acid	TOS	-1.82	5.32	4	2.67	2	1				
Hydroquinone	HYQ	-1.25	5.89	5	4.24	31	1		TBME		
1-hydroxy-2-naphthoic acid	XIN	-1.63	6.02	6	4.21	28	1		ETOH	1-butanol	
Gallic acid	GLL	-2.50	6.18	7	4.59	48	1	THF, ETOH	ETOH	ETOH	
Gentisic acid	GEN	-1.75	6.42	8	4.58	47	1			MeOH	
Saccharin	SAC	-0.20	6.43	9	3.34	12	1				
Caffeine	CAF	-0.16	6.47	10	4.62	49	1				
3,4-dihydroxybenzoic acid	DHB	-1.66	6.50	11	4.38	40	1	MeOH, TBME	MeCN		
Salicylic acid	SAL	-1.15	6.51	12	4.35	38	1	MeOH, THF	toluene	MeOH	
Benzoic acid	BNZ	-0.46	6.68	13	4.23	30	1			MeCN	MeCN

Coformer name	Abbrev.	Computational screening					Experimental results				
		ΔH_{mix} (COSMO quick)	F_{screen} (COSMO quick)	Rank (COSMO)	C_{score} (MC)	Rank (MC)	HTS Type	HTS	LAG	Solvent evaporation	Solvent evaporation
Maltol	MLL	0.06	6.69	14	4.33	37	1				
2-amino-5-methylbenzoic acid	AMB	-0.94	6.71	15	3.88	19	1				
N-hydroxysuccinimide	NHS	0.11	6.74	16	4.07	23	1				
Maleic acid	MLE	-1.40	6.76	17	4.30	35	1				
Fumaric acid	FUM	-1.40	6.76	18	4.82	51	1				
Naphthalene-2-sulfonamide	NSA	-0.12	7.03	19	2.93	5	1-2	THF	METOH		
L-proline	PRO	-0.03	7.12	20	3.29	9	1				
Thymine	THN	0.49	7.12	21	4.41	43	2				
p-toluenesulfonamide	TSA	0.03	7.17	22	2.74	3	1-2	ETOH, MeOH, TBME	ETOH	ETOH	MeCN, ETOH
4-aminosalycylic acid	ASA	-0.99	7.17	23	4.53	46	2				
Ethyl maltol	ETM	0.05	7.20	24	3.26	8	1	TBME, ETOH	ETOH	ETOH, TBME	
Nicotinic acid	NIC	0.08	7.23	25	4.26	33	1	MeOH, ETOH	ETOH	ETOH	
Methyl-4-hydroxybenzoate	MHB	-0.34	7.32	26	3.86	18	1	ETOH, MeOH, TBME	ETOH		MeCN
4-aminobenzoic acid	4ABA	-0.32	7.33	27	4.37	39	1				
Isonicotinamide	INA	0.33	7.47	28	4.40	42	1			ETOH	
Nicotinamide	NCT	-0.34	7.48	29	4.16	25	1		ETOH		MeCN
L-lactic acid	LLA	-0.15	7.51	30	4.21	29	1				
L-pyroglutamic acid	PGL	0.38	7.53	31	3.24	7	2				
Vanillic acid	VAN	-0.60	7.56	32	3.59	15	2				
Cinnamic acid	CIN	-0.08	7.57	33	4.51	45	1	ETOH	ETOH		
Sorbic acid	SOR	-0.05	7.60	34	3.92	21	2				
D-mandelic acid	DMA	-0.56	7.61	35	2.56	1	2				
Vanillin	VNL	0.02	7.67	36	4.06	22	2	MeOH-H2O			

Coformer name	Abbrev.	Computational screening					Experimental results				
		ΔH_{mix} (COSMO quick)	F_{screen} (COSMO quick)	Rank (COSMO)	C_{score} (MC)	Rank (MC)	HTS Type	HTS	LAG	Solvent evaporation	Solvent evaporation
Acetylsalicylic acid	ASS	-0.46	7.70	37	3.58	14	2				
L-tartaric acid	LTA	-1.97	7.73	38	4.32	36	2				
4-hydroxycinnamic acid	HCA	-0.41	7.75	39	4.25	32	2				
Malonic acid	MLO	-0.35	7.82	40	4.27	34	2				
Ethyl gallate	GAE	-1.35	7.84	41	4.09	24	2				
2-oxoglutaric acid	OGL	-1.29	7.89	42	4.16	26	2				
L-alanine	ALA	0.26	7.91	43	4.19	27	2	METOH-H20			
Succinic acid	SUC	-0.73	7.94	44	4.67	50	2				
L-malic acid	MLA	-1.15	8.03	45	3.71	17	2				
Glycine	GLY	0.44	8.09	46	4.44	44	2	MECN			
Trans-aconitic acid	ACA	-1.55	8.15	47	3.54	13	2				
L-valine	VAL	0.19	8.35	48	3.30	10	2	METOH-H2O			
Propyl gallate	GAP	-1.33	8.36	49	3.33	11	2				
N-acetylglycine	ACG	0.41	8.57	50	3.04	6	2				
Ferulic acid	FER	-0.07	8.60	51	3.91	20	2				
Tromethamine	TRO	0.63	9.81	52	4.39	41	1		ETOH		

HTS-1	1	2	3	4	5	6	7	8	9	10	11	12
A	**CILP**	GLL	SAC	MLL	**CILP**	GLL	SAC	MLL	**CILP**	GLL	SAC	MLL
B	ASA	GEN	SAL	MHB	ASA	GEN	SAL	MHB	ASA	GEN	SAL	MHB
C	BES	LLA	TOS	NSA	BES	LLA	TOS	NSA	BES	LLA	TOS	NSA
D	BNZ	MLE	TRO	NCT	BNZ	MLE	TRO	NCT	BNZ	MLE	TRO	NCT
E	AMB	NDS	CAF	PRO	AMB	NDS	CAF	PRO	AMB	NDS	CAF	PRO
F	CIN	XIN	ETM	NHS	CIN	XIN	ETM	NHS	CIN	XIN	ETM	NHS
G	FUM	NIC	HYQ	TSA	FUM	NIC	HYQ	TSA	FUM	NIC	HYQ	TSA
H	DHB	OXA	INA	4ABA	DHB	OXA	INA	4ABA	DHB	OXA	INA	4ABA
Evapor.	Acetone				Methanol				THF			
Slurry	TBME				Methanol				Ethanol			

Figure S1. Layout of the 96-well microtiter plate employed in the "manual" HTS-1 of CILP; The solvents employed in the evaporation and slurry equilibration steps are reported at the bottom of the plate for each replicate. Yellow wells = weak leads - observation of new Raman peaks; Blue wells = strong leads - new Raman spectrum observed. Three control experiments without a conformer were carried out in wells A1, A5, and A9.

HTS-2	1	2	3	4	5	6	7	8	9	10	11	12
A	**CILP**	TAR	ACA	**CILP**	TAR	ACA	**CILP**	TAR	ACA	**CILP**	TAR	ACA
B	NSA	NCA	GAP	NSA	NCA	GAP	NSA	NCA	GAP	NSA	NCA	GAP
C	VAN	MLO	FER	VAN	MLO	FER	VAN	MLO	FER	VAN	MLO	FER
D	SOR	GAE	PGL	SOR	GAE	PGL	SOR	GAE	PGL	SOR	GAE	PGL
E	DMA	OGL	THN	DMA	OGL	THN	DMA	OGL	THN	DMA	OGL	THN
F	TSA	MLA	ALA	TSA	MLA	ALA	TSA	MLA	ALA	TSA	MLA	ALA
G	VLN	SUC	GLY	VLN	SUC	GLY	VLN	SUC	GLY	VLN	SUC	GLY
H	ASS	ACG	VAL	ASS	ACG	VAL	ASS	ACG	VAL	ASS	ACG	VAL
Exp.	Evaporation			Slurry			Slurry			Slurry		
Solvent	Ethanol			Ethanol			Acetonitrile			Methanol - H2O		

Figure S2. Layout of the 96-well microtiter plate employed in the semi-automatic HTS-2 of CILP; 23 coformers were tested under four different conditions: evaporation in ethanol, slurry in ethanol, slurry in acetonitrile and slurry in methanol-water 50%. Yellow wells = weak leads - observation of new PXRD reflections; Blue wells = strong leads -new diffraction pattern observed. Four control experiments without a conformer were carried out in wells A1, A4, A7, and A10.

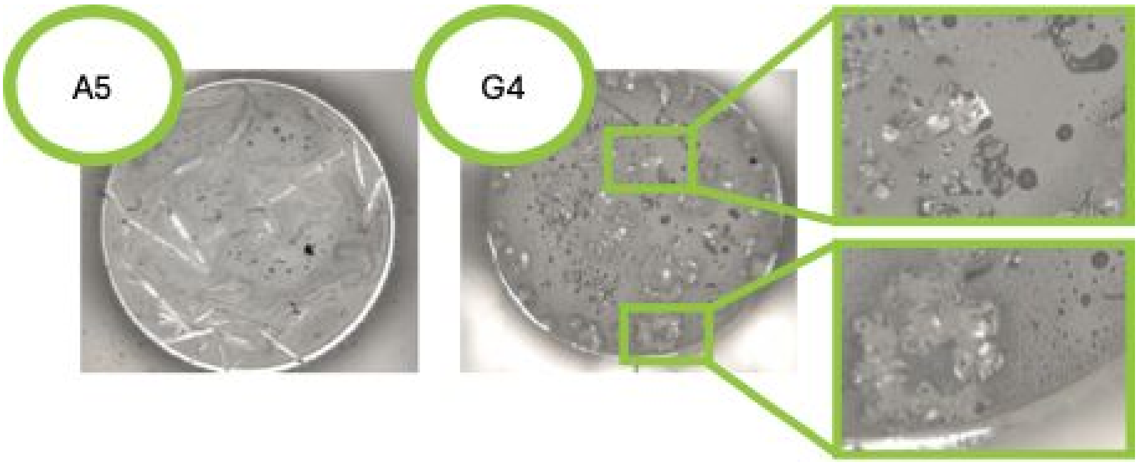

Figure S3. Pictures of the HTS-1 plate wells A5 and G4 collected at the Raman microscope: A5 contains cilnidipine after evaporation in methanol; G4 contains cilnidipine (CILP) and *p*-toluenesulfonamide (TSA) after slurry equilibration in TBME. It is possible to observe the different morphology of the crystals in the two wells.

1.2 Molecular complementarity results: complementarity score

Table S2. Final MC coformer ranking based on the complementarity score (C_{score}) averaged over the 10 cilnidipine conformations. For a simplified representation, the C_{score} reported in the table is already averaged over coformer conformations. *p*-toluenesulfonamide is highlighted in blue.

Coformer name	Cilnidipine conformations										Average Cscore	Rank
	1	2	3	4	5	6	7	8	9	10		
D-mandelic acid	2.60	2.49	3.37	3.04	1.94	1.95	2.53	2.25	2.93	2.46	2.56	1
p-toluenesulfonic acid	2.73	2.62	3.48	3.11	2.05	2.05	2.65	2.34	3.08	2.55	2.67	2
p-toluenesulfonamide	2.70	2.57	3.64	3.27	2.10	2.14	2.68	2.34	3.24	2.71	2.74	3
Benzensulfonic acid	2.88	2.77	3.44	3.26	2.21	2.20	2.80	2.65	2.75	2.59	2.75	4
Naphthalene-2-sulfonamide	2.97	2.87	3.78	3.41	2.30	2.29	2.89	2.59	3.38	2.85	2.93	5
N-acetylglycine	2.89	2.77	3.83	3.46	2.50	2.54	3.01	2.95	3.44	3.00	3.04	6
L-pyroglutamic acid	3.26	3.15	3.82	3.64	2.83	2.83	3.37	3.34	3.11	3.11	3.24	7
Ethyl maltol	3.42	3.31	3.98	3.80	2.75	2.74	3.34	3.04	3.10	3.13	3.26	8
L-proline	3.24	3.27	3.65	3.74	2.97	2.97	3.51	3.48	2.78	3.25	3.29	9
L-valine	3.25	3.24	3.74	3.72	2.94	2.94	3.48	3.45	2.98	3.22	3.30	10
Propyl gallate	3.28	3.16	4.22	3.85	2.69	2.73	3.26	2.92	3.83	3.30	3.33	11
Saccharin	3.37	3.27	3.93	3.75	2.95	2.95	3.49	3.46	3.05	3.23	3.34	12
Trans-aconitic acid	3.54	3.43	4.10	3.92	3.26	3.26	3.64	3.62	3.22	3.38	3.54	13
Acetylsalicylic acid	3.74	3.63	4.30	4.12	3.07	3.06	3.66	3.36	3.42	3.45	3.58	14
Vanillic acid	3.71	3.60	4.27	4.09	3.03	3.03	3.63	3.32	3.81	3.42	3.59	15
Naphthalene-1,5-disulfonic acid	3.76	3.65	4.32	4.14	3.08	3.08	3.68	3.37	3.44	3.47	3.60	16
L-malic acid	3.68	3.57	4.40	4.11	3.12	3.12	3.68	3.50	3.99	3.54	3.67	17
Methyl-4-hydroxybenzoate	3.82	3.70	4.76	4.39	3.23	3.26	3.80	3.46	4.36	3.84	3.86	18
2-amino-5-methylbenzoic_acid	4.02	3.92	4.59	4.41	3.35	3.35	3.95	3.64	3.88	3.74	3.88	19
Ferulic acid	4.05	3.95	4.61	4.43	3.38	3.37	3.97	3.67	3.89	3.76	3.91	20
Sorbic acid	3.88	3.76	4.82	4.45	3.29	3.32	3.86	3.52	4.43	3.90	3.92	21

Coformer name	Cilnidipine conformations										Average Cscore	Rank
	1	2	3	4	5	6	7	8	9	10		
Vanillin	4.22	4.12	4.78	4.60	3.55	3.54	4.14	3.84	3.90	3.93	4.06	22
N-hydroxysuccinimide	4.22	4.12	4.79	4.61	3.55	3.55	4.15	3.84	3.91	3.94	4.07	23
Ethyl gallate	4.18	4.08	4.74	4.57	3.62	3.62	4.11	3.80	4.25	3.90	4.09	24
2-oxoglutaric acid	4.11	3.99	5.06	4.68	3.54	3.58	4.09	3.75	4.66	4.13	4.16	25
Nicotinamide	4.28	4.17	4.84	4.66	3.60	3.60	4.20	3.89	4.41	3.99	4.16	26
L-alanine	4.15	4.18	4.56	4.65	3.88	3.88	4.42	4.39	3.60	4.16	4.19	27
1-hydroxy-2-naphthoic acid	4.32	4.21	4.88	4.70	3.64	3.64	4.24	3.93	4.47	4.03	4.21	28
L-lactic acid	4.18	4.20	4.59	4.68	3.91	3.91	4.44	4.42	3.62	4.19	4.21	29
Benzoic acid	4.34	4.24	4.90	4.73	3.67	3.66	4.27	3.96	4.49	4.06	4.23	30
Hydroquinone	4.40	4.29	4.96	4.78	3.72	3.72	4.32	4.01	4.08	4.11	4.24	31
4-hydroxycinnamic acid	4.21	4.08	5.15	4.78	3.61	3.65	4.19	3.85	4.75	4.23	4.25	32
Nicotinic acid	4.39	4.28	4.95	4.77	3.72	3.71	4.31	4.01	4.35	4.10	4.26	33
Malonic_acid	4.34	4.23	5.07	4.72	3.67	3.66	4.26	3.96	4.67	4.14	4.27	34
Maleic acid	4.35	4.25	5.11	4.74	3.70	3.70	4.28	3.97	4.72	4.19	4.30	35
L-tartaric acid	4.27	4.29	4.72	4.77	4.00	4.00	4.54	4.51	3.84	4.28	4.32	36
Maltol	4.48	4.38	5.04	4.87	3.81	3.81	4.41	4.10	4.16	4.20	4.33	37
Salicylic acid	4.51	4.40	5.07	4.89	3.84	3.83	4.43	4.13	4.19	4.22	4.35	38
4-aminobenzoic_acid	4.37	4.26	5.24	4.87	3.71	3.75	4.29	3.99	4.85	4.32	4.37	39
3,4-dihydroxybenzoic acid	4.54	4.43	5.10	4.92	3.87	3.86	4.46	4.16	4.22	4.25	4.38	40
Tromethamine	4.27	4.30	4.68	4.76	4.41	4.42	4.53	4.51	3.71	4.27	4.39	41
Isonicotinamide	4.55	4.45	5.11	4.93	3.88	3.87	4.47	4.17	4.32	4.26	4.40	42
Thymine	4.56	4.46	5.12	4.95	3.89	3.88	4.49	4.18	4.24	4.28	4.41	43
Glycine	4.56	4.46	5.12	4.95	3.89	3.89	4.49	4.18	4.55	4.28	4.44	44
Cinnamic acid	4.47	4.36	5.40	5.03	3.87	3.90	4.44	4.10	5.01	4.48	4.51	45
4-aminosalycylic acid	4.69	4.58	5.25	5.07	4.02	4.01	4.61	4.31	4.37	4.40	4.53	46

Coformer name	Cilnidipine conformations										Average Cscore	Rank
	1	2	3	4	5	6	7	8	9	10		
Gentisic acid	4.74	4.63	5.30	5.12	4.07	4.06	4.66	4.36	4.42	4.45	4.58	47
Gallic acid	4.75	4.64	5.31	5.13	4.08	4.07	4.67	4.37	4.43	4.46	4.59	48
Caffeine	4.78	4.67	5.34	5.16	4.11	4.10	4.70	4.40	4.46	4.49	4.62	49
Succinic acid	4.63	4.50	5.57	5.20	4.03	4.07	4.61	4.27	5.17	4.64	4.67	50
Fumaric acid	4.78	4.65	5.72	5.35	4.18	4.22	4.76	4.42	5.32	4.79	4.82	51
Oxalic acid	5.34	5.23	5.90	5.72	4.66	4.66	5.26	4.95	5.02	5.05	5.18	52

1.3 Molecular complementarity results: hit rate calculation using default Mercury settings

Table S3. Final MC results obtained with the MC default settings available in Mercury. FAIL = coformer has not passed the MC analysis with the API conformation (this failure occurs when the difference between the API and the coformer in at least one of the molecular descriptors exceeds the cut-off value). Hit rate represents the percentage of API conformations that successfully pass the MC analysis with the coformer. If the hit rate is 0%, it means that none of the API conformations passed the MC analysis with the coformer, indicating that the API and coformer are unlikely to form co-crystals. n.a. = not applicable.

Coformer name	Cilnidipine conformations										Hit rate (%)	Rank
	1	2	3	4	5	6	7	8	9	10		
D-mandelic acid	FAIL	FAIL	FAIL	FAIL	FAIL	FAIL	FAIL	FAIL	FAIL	FAIL	0	n.a
p-toluenesulfonic acid	FAIL	FAIL	FAIL	FAIL	FAIL	FAIL	FAIL	FAIL	FAIL	FAIL	0	n.a
p-toluenesulfonamide	FAIL	FAIL	FAIL	FAIL	FAIL	FAIL	FAIL	FAIL	FAIL	FAIL	0	n.a
Benzensulfonic acid	FAIL	FAIL	FAIL	FAIL	FAIL	FAIL	FAIL	FAIL	FAIL	FAIL	0	n.a
Naphthalene-2-sulfonamide	FAIL	FAIL	FAIL	FAIL	FAIL	FAIL	FAIL	FAIL	FAIL	FAIL	0	n.a
N-acetylglycine	FAIL	FAIL	FAIL	FAIL	FAIL	FAIL	FAIL	FAIL	FAIL	FAIL	0	n.a
L-pyroglutamic acid	FAIL	FAIL	FAIL	FAIL	FAIL	FAIL	FAIL	FAIL	FAIL	FAIL	0	n.a
Ethyl maltol	FAIL	FAIL	FAIL	FAIL	FAIL	FAIL	FAIL	FAIL	FAIL	FAIL	0	n.a
L-proline	FAIL	FAIL	FAIL	FAIL	FAIL	FAIL	FAIL	FAIL	FAIL	FAIL	0	n.a

Coformer name	Cilnidipine conformations										Hit rate (%)	Rank
	1	2	3	4	5	6	7	8	9	10		
L-valine	FAIL	FAIL	FAIL	FAIL	FAIL	FAIL	FAIL	FAIL	FAIL	FAIL	0	n.a
Propyl gallate	FAIL	FAIL	FAIL	FAIL	FAIL	FAIL	FAIL	FAIL	FAIL	FAIL	0	n.a
Saccharin	FAIL	FAIL	FAIL	FAIL	FAIL	FAIL	FAIL	FAIL	FAIL	FAIL	0	n.a
Trans-aconitic acid	FAIL	FAIL	FAIL	FAIL	FAIL	FAIL	FAIL	FAIL	FAIL	FAIL	0	n.a
Acetylsalicylic acid	FAIL	FAIL	FAIL	FAIL	FAIL	FAIL	FAIL	FAIL	FAIL	FAIL	0	n.a
Vanillic acid	FAIL	FAIL	FAIL	FAIL	FAIL	FAIL	FAIL	FAIL	FAIL	FAIL	0	n.a
Naphthalene-1,5-disulfonic acid	FAIL	FAIL	FAIL	FAIL	FAIL	FAIL	FAIL	FAIL	FAIL	FAIL	0	n.a
L-malic acid	FAIL	FAIL	FAIL	FAIL	FAIL	FAIL	FAIL	FAIL	FAIL	FAIL	0	n.a
Methyl-4-hydroxybenzoate	FAIL	FAIL	FAIL	FAIL	FAIL	FAIL	FAIL	FAIL	FAIL	FAIL	0	n.a
2-amino-5-methylbenzoic_acid	FAIL	FAIL	FAIL	FAIL	FAIL	FAIL	FAIL	FAIL	FAIL	FAIL	0	n.a
Ferulic acid	FAIL	FAIL	FAIL	FAIL	FAIL	FAIL	FAIL	FAIL	FAIL	FAIL	0	n.a
Sorbic acid	FAIL	FAIL	FAIL	FAIL	FAIL	FAIL	FAIL	FAIL	FAIL	FAIL	0	n.a
Vanillin	FAIL	FAIL	FAIL	FAIL	FAIL	FAIL	FAIL	FAIL	FAIL	FAIL	0	n.a
N-hydroxysuccinimide	FAIL	FAIL	FAIL	FAIL	FAIL	FAIL	FAIL	FAIL	FAIL	FAIL	0	n.a
Ethyl gallate	FAIL	FAIL	FAIL	FAIL	FAIL	FAIL	FAIL	FAIL	FAIL	FAIL	0	n.a
2-oxoglutaric acid	FAIL	FAIL	FAIL	FAIL	FAIL	FAIL	FAIL	FAIL	FAIL	FAIL	0	n.a
Nicotinamide	FAIL	FAIL	FAIL	FAIL	FAIL	FAIL	FAIL	FAIL	FAIL	FAIL	0	n.a
L-alanine	FAIL	FAIL	FAIL	FAIL	FAIL	FAIL	FAIL	FAIL	FAIL	FAIL	0	n.a
1-hydroxy-2-naphthoic acid	FAIL	FAIL	FAIL	FAIL	FAIL	FAIL	FAIL	FAIL	FAIL	FAIL	0	n.a
L-lactic acid	FAIL	FAIL	FAIL	FAIL	FAIL	FAIL	FAIL	FAIL	FAIL	FAIL	0	n.a
Benzoic acid	FAIL	FAIL	FAIL	FAIL	FAIL	FAIL	FAIL	FAIL	FAIL	FAIL	0	n.a
Hydroquinone	FAIL	FAIL	FAIL	FAIL	FAIL	FAIL	FAIL	FAIL	FAIL	FAIL	0	n.a
4-hydroxycinnamic acid	FAIL	FAIL	FAIL	FAIL	FAIL	FAIL	FAIL	FAIL	FAIL	FAIL	0	n.a
Nicotinic acid	FAIL	FAIL	FAIL	FAIL	FAIL	FAIL	FAIL	FAIL	FAIL	FAIL	0	n.a
Malonic_acid	FAIL	FAIL	FAIL	FAIL	FAIL	FAIL	FAIL	FAIL	FAIL	FAIL	0	n.a

Coformer name	Cilnidipine conformations										Hit rate (%)	Rank
	1	2	3	4	5	6	7	8	9	10		
Maleic acid	FAIL	FAIL	FAIL	FAIL	FAIL	FAIL	FAIL	FAIL	FAIL	FAIL	0	n.a
L-tartaric acid	FAIL	FAIL	FAIL	FAIL	FAIL	FAIL	FAIL	FAIL	FAIL	FAIL	0	n.a
Maltol	FAIL	FAIL	FAIL	FAIL	FAIL	FAIL	FAIL	FAIL	FAIL	FAIL	0	n.a
Salicylic acid	FAIL	FAIL	FAIL	FAIL	FAIL	FAIL	FAIL	FAIL	FAIL	FAIL	0	n.a
4-aminobenzoic_acid	FAIL	FAIL	FAIL	FAIL	FAIL	FAIL	FAIL	FAIL	FAIL	FAIL	0	n.a
3,4-dihydroxybenzoic acid	FAIL	FAIL	FAIL	FAIL	FAIL	FAIL	FAIL	FAIL	FAIL	FAIL	0	n.a
Tromethamine	FAIL	FAIL	FAIL	FAIL	FAIL	FAIL	FAIL	FAIL	FAIL	FAIL	0	n.a
Isonicotinamide	FAIL	FAIL	FAIL	FAIL	FAIL	FAIL	FAIL	FAIL	FAIL	FAIL	0	n.a
Thymine	FAIL	FAIL	FAIL	FAIL	FAIL	FAIL	FAIL	FAIL	FAIL	FAIL	0	n.a
Glycine	FAIL	FAIL	FAIL	FAIL	FAIL	FAIL	FAIL	FAIL	FAIL	FAIL	0	n.a
Cinnamic acid	FAIL	FAIL	FAIL	FAIL	FAIL	FAIL	FAIL	FAIL	FAIL	FAIL	0	n.a
4-aminosalycylic acid	FAIL	FAIL	FAIL	FAIL	FAIL	FAIL	FAIL	FAIL	FAIL	FAIL	0	n.a
Gentisic acid	FAIL	FAIL	FAIL	FAIL	FAIL	FAIL	FAIL	FAIL	FAIL	FAIL	0	n.a
Gallic acid	FAIL	FAIL	FAIL	FAIL	FAIL	FAIL	FAIL	FAIL	FAIL	FAIL	0	n.a
Caffeine	FAIL	FAIL	FAIL	FAIL	FAIL	FAIL	FAIL	FAIL	FAIL	FAIL	0	n.a
Succinic acid	FAIL	FAIL	FAIL	FAIL	FAIL	FAIL	FAIL	FAIL	FAIL	FAIL	0	n.a
Fumaric acid	FAIL	FAIL	FAIL	FAIL	FAIL	FAIL	FAIL	FAIL	FAIL	FAIL	0	n.a
Oxalic acid	FAIL	FAIL	FAIL	FAIL	FAIL	FAIL	FAIL	FAIL	FAIL	FAIL	0	n.a

2 Cilnidipine (CILP)

2.1 TG-FTIR

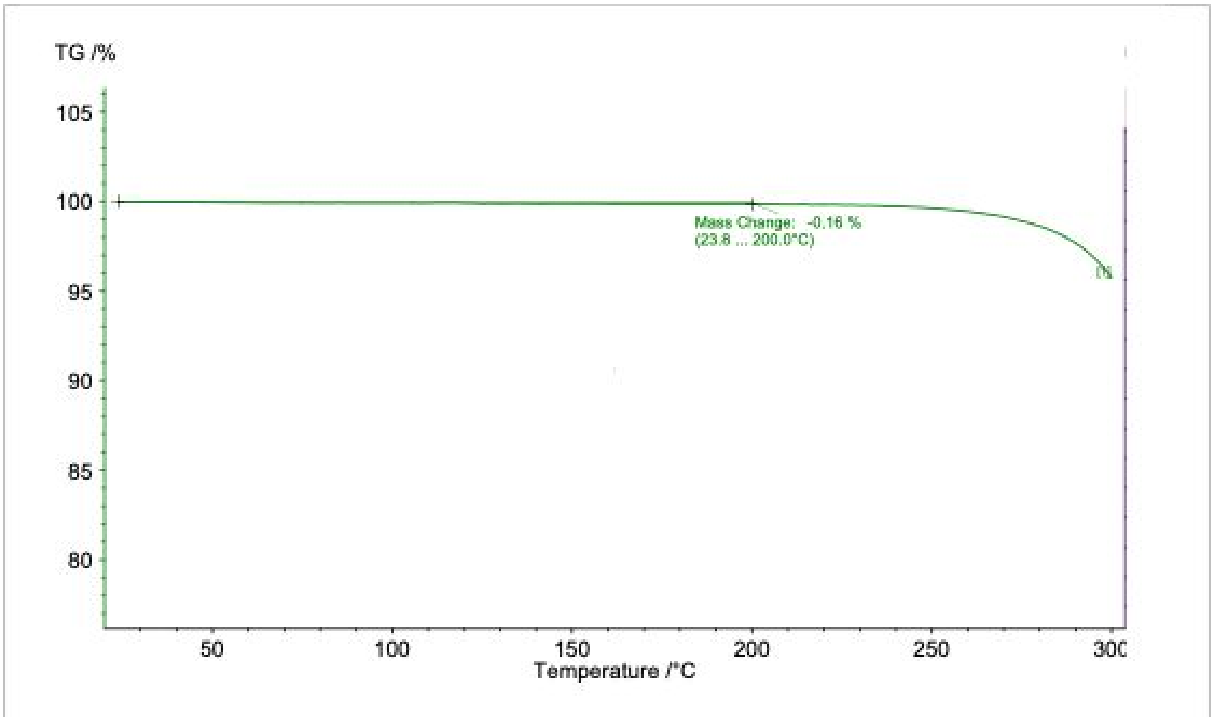

Figure S4. Thermogravimetric analysis of crystalline cilnidipine performed with a heating rate of 10 K/min. The thermogram is reported as percentage of weight loss. The sample is anhydrous.

2.2 Proton Nuclear Magnetic Resonance (^{1}H-NMR)

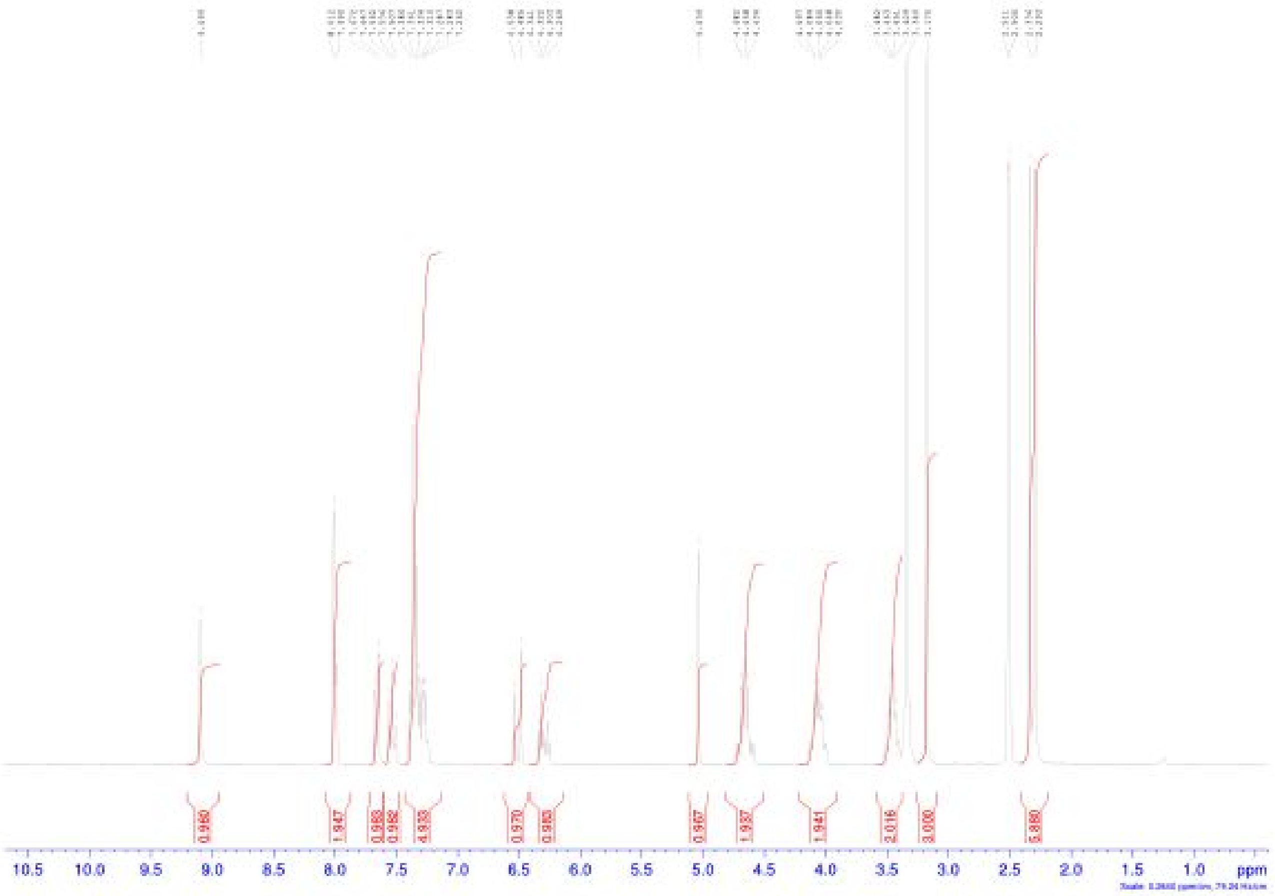

Figure S5. Proton NMR spectrum of purchased cilnidipine collected in deuterated DMSO. Peak integration is referred to the singlet at 3.18 ppm corresponding to three protons of cilnidipine.

3 Cilnidipine – *p*-Toluenesulfonamide 1:1 Co-crystal (CILP-TSA)

3.1 TG-FTIR

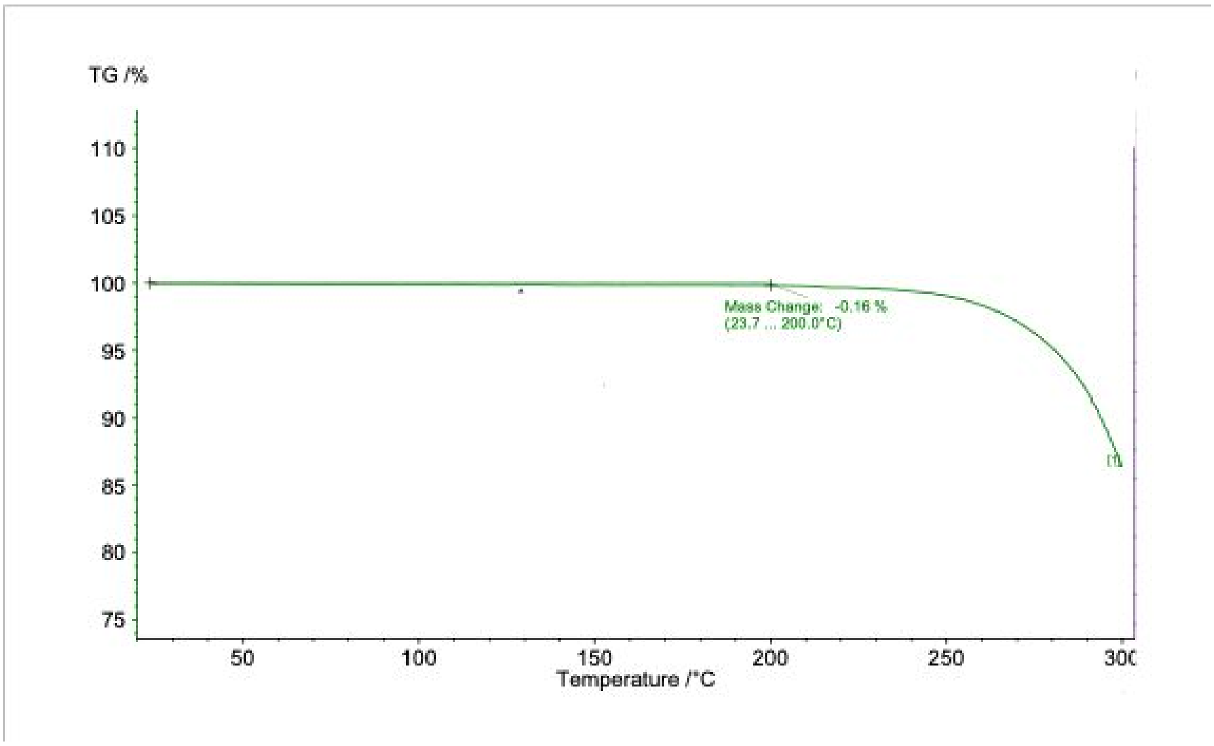

Figure S6. Thermogravimetric analysis of CILP-TSA performed with a heating rate of 10 K/min. The thermogram is reported as percentage of weight loss. The sample is anhydrous.

3.2 Proton Nuclear Magnetic Resonance (^{1}H-NMR)

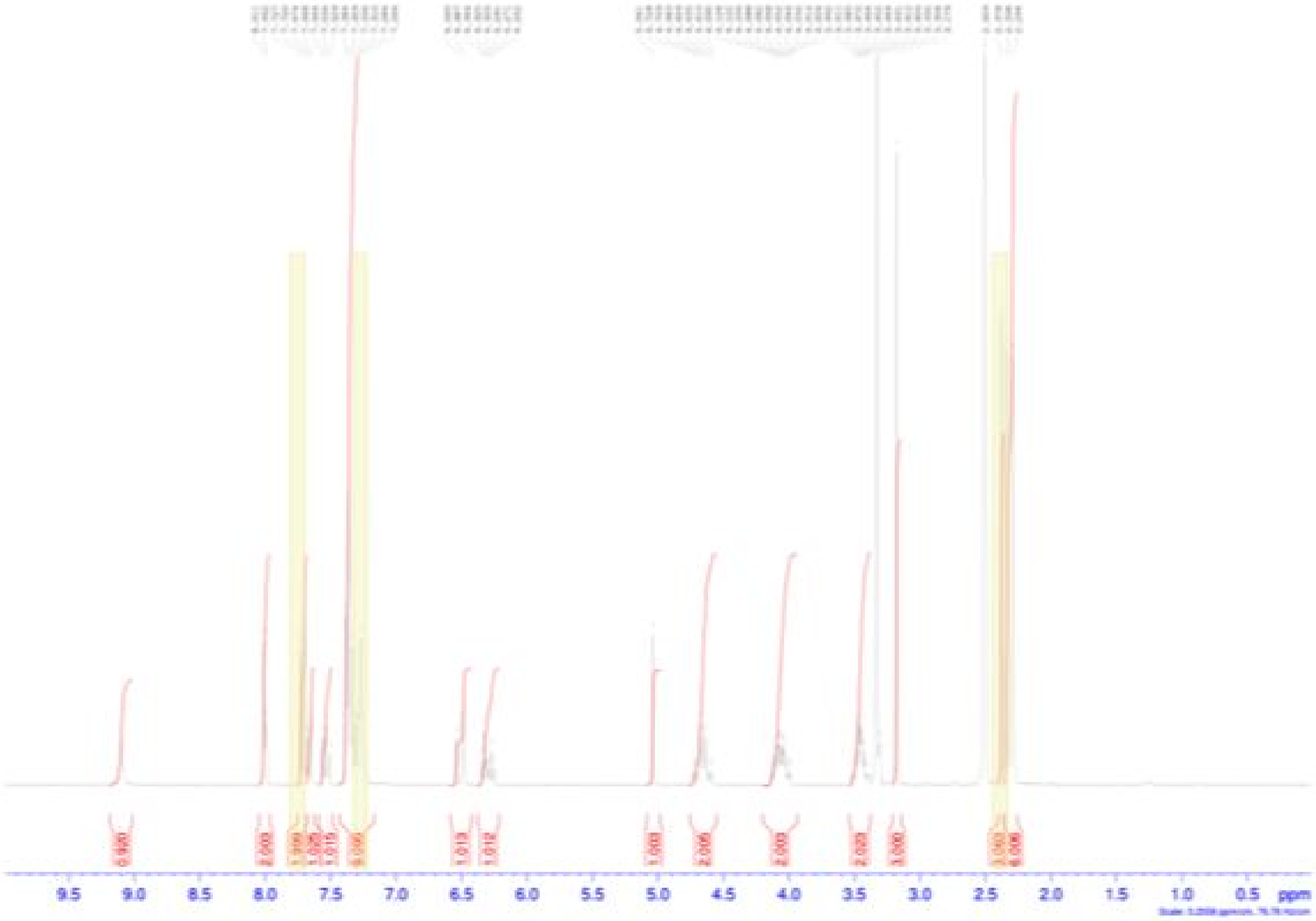

Figure S7. Proton NMR spectrum of prepared CILP-TSA co-crystal collected in deuterated DMSO. Proton signals and integrations of *p*-toluenesulfonamide (TSA) are highlighted in yellow. The integration suggests a 1:1 stoichiometry of CILP and TSA

3.3 Melting point and enthalpy of fusion co-crystal

Table S4. Melting temperature (T_m) and enthalpy of fusion (ΔH_{fus}) of CILP, TSA and CILP-TSA co-crystal measured by DSC. Concentrations of CILP and TSA determined by dissolving a sample of co-crystal in acetonitrile.

Crystal form	T_m (°C)	ΔH_{fus} (kJ/mol)
CILP	105.8	45.5
TSA	137.7	23.4
CILP-TSA	122.6	62.5

3.4 UHPLC-UV Co-crystal

The stoichiometry suggested by ^{1}H-NMR was further corroborated by UHPLC-UV analysis of the co-crystal. 9.5 mg of co-crystal were dissolved in 25 mL of acetonitrile; after appropriate dilution, the sample was tested by UHPLC-UV to determine the concentrations of both TSA and CILP. The gradient method reported in the material and method section of this manuscript was used. The final concentrations of the two components are reported in the table below. The molar concentrations confirm a 1:1 stoichiometric ratio of the two components (difference % = 2.5%).

Table S5. Concentrations of CILP and TSA determined by dissolving a sample of co-crystal in acetonitrile.

Component	MW (g/mol)	Detection λ (nm)	Retention time (min)	Conc. (µg/mL)	Conc. (µM)
CILP	492.5	240	3.98	13.5	27.3
TSA	171.2	224	1.55	4.8	28.0

4 Co-crystal Dissolution Studies

The amount of co-crystal dissolved was calculated by the ratio of dissolved coformer to the total amount of coformer initially added (2.8 mg). Due to the poor wettability of the powder, which tended to float on the top of the dissolution medium and to aggregate, only 36% of

co-crystal dissolved in blank FaSSIF after 24 hours. In the case of FaSSIF, the co-crystals dissolved almost completely after 24 hours and only a fine pale-yellow powder (belonging to CILP) was observed in the dissolution medium. However, few mg of co-crystal powder got stuck on the walls of the container during dissolution and could not be dissolved because they were not in contact with the medium.

Table S6. Amount of dissolved co-crystal in Blank FaSSIF and FaSSIF expressed in % (n=3, mean ± standard deviation)

Medium	30 min	90 min	4 h	8 h	24 h
Blank FaSSIF	4.7 ± 1.0	7.6 ± 11.7	11.7 ± 1.4	17.9 ± 2.3	35.7 ± 1.0
FaSSIF	21.6 ± 0.9	43.1 ± 4.8	66.4 ± 14.1	75.6 ± 7.7	92.0 ± 4.5
